# A systematic review and network meta-analysis of first-line immune checkpoint inhibitor combination therapies in patients with advanced non-squamous non-small cell lung cancer

**DOI:** 10.3389/fimmu.2022.948597

**Published:** 2022-10-26

**Authors:** Taihang Shao, Mingye Zhao, Leyi Liang, Wenxi Tang

**Affiliations:** ^1^ Center for Pharmacoeconomics and Outcomes Research, China Pharmaceutical University, Nanjing, China; ^2^ Department of Public Affairs Management, School of International Pharmaceutical Business, China Pharmaceutical University, Nanjing, China

**Keywords:** non-small cell lung cancer, non-squamous, immune checkpoint inhibitor combination therapies, Royston–Parmar model, restricted mean survival time, network meta-analysis

## Abstract

**Introduction:**

Clinical evidence suggests that first-line immune checkpoint inhibitor (ICI) combination therapies can improve survival in patients with advanced non-squamous non-small cell lung cancer (nsq-NSCLC). However, the optimal strategy remains unknown without a systematic comparison of their long-term effects.

**Methods:**

We performed a systematic review and network meta-analysis by retrieving up-to-date literature from PubMed^®^ (National Library of Medicine, Bethesda, MD, USA), Embase^®^ (Elsevier, Amsterdam, Netherlands), MEDLINE^®^ (National Library of Medicine), ClinicalTrials.gov (National Library of Medicine), and major international conference publications. Published studies and abstracts comparing first-line ICI combination therapies with other treatments for patients with advanced nsq-NSCLC were included. Restricted mean survival time (RMST) was measured over 12 months for progression-free survival (PFS) and 18 months for overall survival (OS), and the Royston–Parmar model was used to extrapolate and compare data for the long-term outcomes.

**Results:**

We included a total of 11 trials involving 12 therapies and 6,130 patients. Pembrolizumab plus chemotherapy exhibited the best overall survival (OS) benefit at both 18 and 60 months [RMST = 2.95, 95% confidence interval (CI) 1.96 to 3.97; life-years gained over a 5-year period = 2.18 years]. Nivolumab plus bevacizumab plus chemotherapy was found to present the best progression-free survival (PFS) benefit at 12 months (RMST 3.02, 95% CI 2.11 to 3.91), whereas atezolizumab plus bevacizumab plus chemotherapy showed the best PFS benefit at 36 months (life-years gained over 3 years = 1.22 years). Subgroup analyses showed that among patients with programmed death-ligand 1 (PD-L1) expression ≥ 50%, atezolizumab plus chemotherapy and nivolumab plus ipilimumab resulted in superior OS benefits at 18 and 60 months, respectively. Among patients with PD-L1 expression< 1%, pembrolizumab plus chemotherapy was associated with OS benefits at both 18 and 60 months. Sintilimab plus chemotherapy was associated with relatively fewer grade ≥ 3 adverse events than other ICI combination therapies.

**Conclusion:**

Our results show that ICI combination therapies showed better survival benefits than chemotherapy. Pembrolizumab plus chemotherapy could provide the best OS benefits to patients with advanced nsq-NSCLC, whereas atezolizumab plus bevacizumab plus chemotherapy could bring the best PFS benefits. The optimal ICI combination therapy varies depending on PD-L1 expression level.

**Systematic Review Registration:**

https://www.crd.york.ac.uk/PROSPERO/display_record.php?RecordID=325005, identifier CRD42022325005.

## Introduction

Lung cancer is the second most commonly diagnosed cancer and the leading cause of cancer death worldwide. Non-small cell lung cancer (NSCLC) accounts for approximately 85% of all reported cases of lung cancer (1, 2). More than half of patients with NSCLC are found to have cancer of non-squamous histology (3). Platinum-doublet chemotherapy has for decades been the standard first-line treatment for patients with advanced nsq-NSCLC who lack targetable genetic alterations, but with a median overall survival (OS) of less than 12 months (4).

In recent years, the emergence of immune checkpoint inhibitors (ICIs) has drastically altered the landscape of cancer treatment. ICIs, which include cytotoxic T-lymphocyte-associated protein 4 (CTLA-4), programmed cell death protein 1 (PD-1), and programmed cell death ligand 1 (PD-L1), have been proven in multiple clinical trials (5–8) to provide additional progression-free survival (PFS) and OS benefits. Specifically, several randomized phase III trials have shown that combining ICIs (PD-1/PD-L1) with platinum-based chemotherapy as the first-line therapy in patients non-squamous NSCLC (nsq-NSCLC) provides significantly improved greater survival benefits than chemotherapy alone. Given the encouraging evidence, nivolumab, ipilimumab, pembrolizumab, and atezolizumab were, between 2016 and 2018, approved by the US Food and Drug Administration (FDA) for the first-line treatment of nsq-NSCLC (9). In China, pembrolizumab, atezolizumab (which came from an international manufacturer), and camrelizumab, tislelizumab, sintilimab, and sugemalimab (which came from a local manufacturer) were approved by the Chinese National Medical Products Administration (NMPA) between 2020 and 2022 (10). In addition to these novel drugs, the combination of these ICIs is also considered in clinical practice, as combinations may enhance antitumor activity and offer incremental clinical benefits (11). Recently, a series of clinical trials have explored the effect of ICI combination therapies including ICIs plus chemotherapy and combinations of two or more ICIs (12).

However, despite advances in treatment, we are unable to determine which combination therapy achieves the greatest long-term survival benefits owing to a lack of evidence from head-to-head trials. Several network meta-analyses (NMAs) have indirectly compared the clinical benefits of combination therapies (13–16). However, these indirect comparisons were based on the assumption of proportional hazards (PHs), which can only compare the benefits only during the follow-up period of trials. A PH model does not allow for precise longer-term extrapolation, and the synthesis is constrained by the fact that differences are assumed to be consistent across trials and to be independent of differences in absolute survival (17, 18). Therefore, there is a lack of reliable evidence of the long-term clinical benefits of ICIs.

This study aimed to evaluate and compare both the short-term and long-term effects of all currently available first-line ICI combination therapies in patients with advanced nsq-NSCLC. An adjusted indirect comparison on the basis of a Bayesian framework was conducted under the non-PH assumption. Subgroup analyses were also carried out to provide more precise evidence for patients with different PD-L1 expression levels.

## Materials and methods

This indirect comparison was performed in accordance with the Preferred Reporting Items for Systematic Reviews and Meta-Analysis (PRISMA) extension statement for NMAs (19). We used the Bayesian approach to make indirect comparisons between treatments that have not yet been directly compared through clinical trials. The protocol for this study was registered in PROSPERO as CRD42022325005. Codes and data for recreating this analysis are available from GitHub (https://github.com/TaihangShao/nsq_scope_NMA; GitHub, Inc., San Francisco, CA, USA).

### Data sources

We systematically searched databases including PubMed^®^ (National Library of Medicine, Bethesda, MD, USA), Embase^®^ (Elsevier, Amsterdam, Netherlands), MEDLINE^®^ (National Library of Medicine, Bethesda, MD, USA), and ClinicalTrials.gov (National Library of Medicine), for relevant studies published until 10 April 2022. We also searched for conference abstracts for the most up-to-date parameters, including the American Association for Cancer Research, the American Society of Clinical Oncology, the European Society for Medical Oncology, the Chinese Society of Clinical Oncology, the World Conference on Lung Cancer, and the European Lung Cancer Congress, which all took place between 2020 and 2022. The keywords used for searching were “non-small-cell lung cancer, randomized controlled trial, phase III, immune checkpoint inhibitors, immunotherapy, PD-1, PD-L1, CTLA-4, pembrolizumab, atezolizumab, nivolumab, ipilimumab, camrelizumab, tislelizumab, sintilimab, toripalimab, cemiplimab, and sugemalimab.” Detailed information on search strategies can be found in **Supplementary Table 1**.

### Inclusion and exclusion criteria

#### Inclusion criteria

1. Study types: phase III randomized controlled trials.
2. Patients: patients with advanced nsq-NSCLC (stages IIIB–IV) confirmed either histologically or cytologically without targetable genetic alterations.
3. Interventions: first-line treatment with ICIs combined with other treatments, including ICIs plus chemotherapy, ICIs plus anti-angiogenesis drugs, and ICI + ICI combinations.
4. Chemotherapy: platinum-based chemotherapy, including carboplatin/cisplatin plus pemetrexed/paclitaxel/nab-paclitaxel.
5. Efficacy: report of at least one Kaplan–Meier (KM) curve of the indicators OS or PFS. OS was defined as the time from randomization until death from any cause. PFS was defined as the time from randomization to disease progression or death from any cause.
6. Safety: adverse events (AEs) of any grade or AEs of grade ≥ 3.

#### Exclusion criteria

1. Articles relating to trials already included but reporting older results, e.g. of the three articles related to the Keynote189 trial that showed up in our search, we included only the latest one, published in 2021 (20–22).
2. Studies with ambiguous clinical outcomes, e.g., abstracts with no clinical outcomes reported.

All retrieved articles were imported into NoteExpress (version 3.2.0.7535; Aegean Software Corp., Beijing, China). Two independent researchers (Shao and Zhao) screened the literature for inclusion. Titles and abstracts were first screened. Then, the full text of literature selected for inclusion was evaluated. Finally, we checked that the articles included reported the most up-to-date data from each relevant trial.

### Data extraction

Data were extracted independently by two researchers (Shao and Zhao). Extracted data relating to clinical characteristics included the trial name, first author, publication sources, year of publication, National Clinical Trial (NCT) number, sample size, age, sex, ethnicity, smoking status, cancer histologic type, PD-L1 expression, and Eastern Cooperative Oncology Group (ECOG) performance status score. Clinical outcomes extracted included OS and PFS, KM curves of OS and PFS, AEs of any grade, and grade ≥ 3 AEs [hazard ratios (HRs) with corresponding 95% confidence intervals (CIs)].

### Quality assessment

The quality of included studies was assessed using the Cochrane risk-of-bias tool for randomized controlled trials (RCTs) and the following aspects were assessed: random sequence generation, allocation concealment, blinding of outcome assessors, completeness of outcome data, selective outcome reporting, and other potential biases (23). All six aspects were evaluated as (1) low risk of bias, (2) unknown risk of bias, or (3) high risk of bias. A high-quality study was defined as one in which more than four aspects were considered to have a low risk of bias. We used the Egger regression test with a funnel plot to evaluate the publication bias, and a *p*-value of< 0.10 was considered to indicate significant asymmetry and publication bias.

### Statistical analysis

We carried out an indirect comparison using a Bayesian framework. The primary outcomes were OS and PFS, and the secondary outcomes were AEs of any grade and grade ≥ 3 AEs. Restricted mean survival time (RMST) was selected as the short-term measure of OS and PFS, and life-years gained were selected as the long-term outcome (17, 24). Odds ratios (ORs) with 95% CIs were used as the effect sizes for AEs of any grade and grade ≥ 3 AEs.

Probabilities of OS and PFS were extracted from KM curves using GetData Graph Digitizer software version 2.24 (GetData Pty Ltd., Kogarah, Australia). We followed the method of Guyot et al. (25) to reconstruct estimates of individual patient data (IPD) over the period of the clinical trial. To compare short-term effectiveness, we compared the RMSTs in the shortest period of follow-up as reported in the selected trials to capture the survival benefit on an equal basis, that is, RMST of 18 months for OS as reported in the CameL study and of 12 months for PFS as reported in the Rationale 304 study. We first calculated the RMST in each arm, then used a fixed-effects NMA model to estimate the difference in RMST between each treatment and the reference treatment. Forest plots and rank plots were used to visualize the results. To compare the long-term effectiveness, we first examined the assumption of PHs for each trial using log-cumulated hazard plots and Schoenfeld residual tests (26, 27). As the PH assumption did not hold (nearly all studies violated the PH assumption based on intersecting lines on log-cumulated hazard plots), we did an indirect comparison under the assumption of non-proportional hazard. The results of the PH test are shown in **Supplementary Figures 1** and **2**. We used the Royston–Parmar flexible parametric model for extrapolation and indirect comparison because it has been shown to perform better than other parametric models, including a pairwise exponential model and fractional polynomial models (28). Royston–Parmar models with non-proportional hazard assumption were defined as follows:


$$Ln\{H(t|x_{ij})\}=s_{j}(ln(t_{i}))+\beta x_{i}+\alpha x_{i}(ln(t_{i}))$$

$$s_{j}(ln(t_{i}))=\gamma_{1}+\gamma_{1}u_{0}(ln(t_{i}))+\cdot \cdot \cdot \gamma_{p+2}u_{p}(ln(t_{i}))$$

where *Ln*{*H*(*t*|*x_ij_*)} is the log-cumulative hazard and *s_j_* (*ln*(*t_i_*)) is the spline function. In *βx_i_*, *x_i_* is the PH model treatment indicator for patient *i* and β is the coefficient. *αx_i_* (*ln*(*t_i_*)) are the non-PH model treatment indicators. *u_p_* (*ln*(*t_i_*)) is the basic function and γ is its coefficient. Details of the Royston–Parmar model can be found in Freeman and Carpenter (29) and Royston and Parmar (30).

We used R (The R Foundation for Statistical Computing, Vienna, Austria) and WinBUGS (MRC Biostatistics Unit, Cambridge, UK) to implement this indirect comparison. Reference chemotherapy was set as the chemotherapy regimen used in the Keynote-189 study, unless otherwise specified, because Keynote-189 provided final data for all subgroups. For OS and PFS, we used a fixed treatment-effect NMA model with three independent Markov chains running 5,000 burn-ins and 10,000 sample iterations per chain simultaneously using one step-size iteration. γ and β were fitted using non-informative normal prior distributions. Survival plots were used for visual inspection for of fit and extrapolation. We ranked the treatments based on OS at 5 years and PFS at 3 years. We chose these time periods because extrapolation for a longer period might not reflect clinical practice. In addition, owing to the occurrence of plateaus at the tail of KM curves, overextrapolation might also lead to significant overestimation of survival benefits (31, 32). For AEs, we used R package gemtc to summarize the results. The convergence of the model was judged and visualized using the Brooks–Gelman–Rubin method (33). As there was no direct comparison, consistency could not be tested in this study. Heterogeneity was tested only in the case of studies comparing atezolizumab plus chemotherapy with chemotherapy alone, since there were two studies of this comparison. Accordingly, we performed a pairwise IPD meta-analysis to assess heterogeneity within each comparison using a Cox model (34). HRs were selected as outcome indicators, and Cochran’s *Q* statistic and forest plots were used to show heterogeneity (29). Furthermore, we carried out subgroup analyses on trials that targeted both ICIs recommended by the National Comprehensive Cancer Network (NCCN) as well as different PD-L1 expression levels to explore the potential heterogeneity in this indirect comparison. Five ICI combination therapies recommended by the NCCN clinical practice guidelines because of the availability of mature data OS were included in our subgroup analysis: nivolumab plus ipilimumab (niv + ipi), atezolizumab plus chemotherapy (ate + che), nivolumab plus ipilimumab plus chemotherapy (niv + ipi + che), atezolizumab plus bevacizumab plus chemotherapy (ate + bev + che), and pembrolizumab plus chemotherapy (pem + che). For the subgroup analysis of PD-L1 expression levels, owing to the limited availability of data, we divided patients into only two subgroups: patients with PD-L1 expression level< 1% and patients with PD-L1 expression level ≥ 50%. The same methods as the base-case analysis were used when performing the subgroup analyses.

## Results

### Study selection and characteristics of included studies

**Figure 1:**
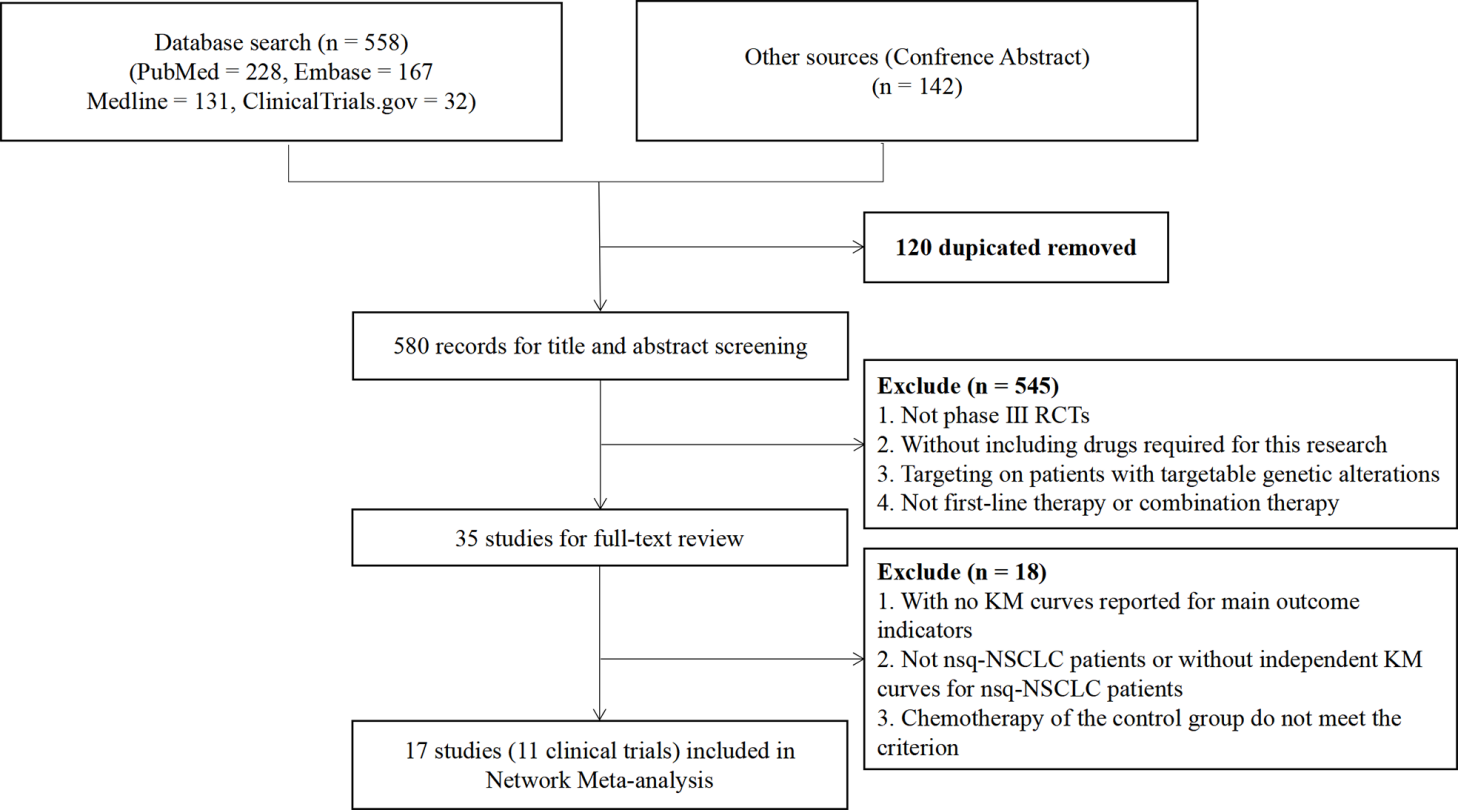
Preferred Reporting Items for Systematic Reviews and Meta-Analyses (PRISMA) flow diagram showing the process of literature searching and selection. The process followed the PRISMA guidelines.

**Figure 2:**
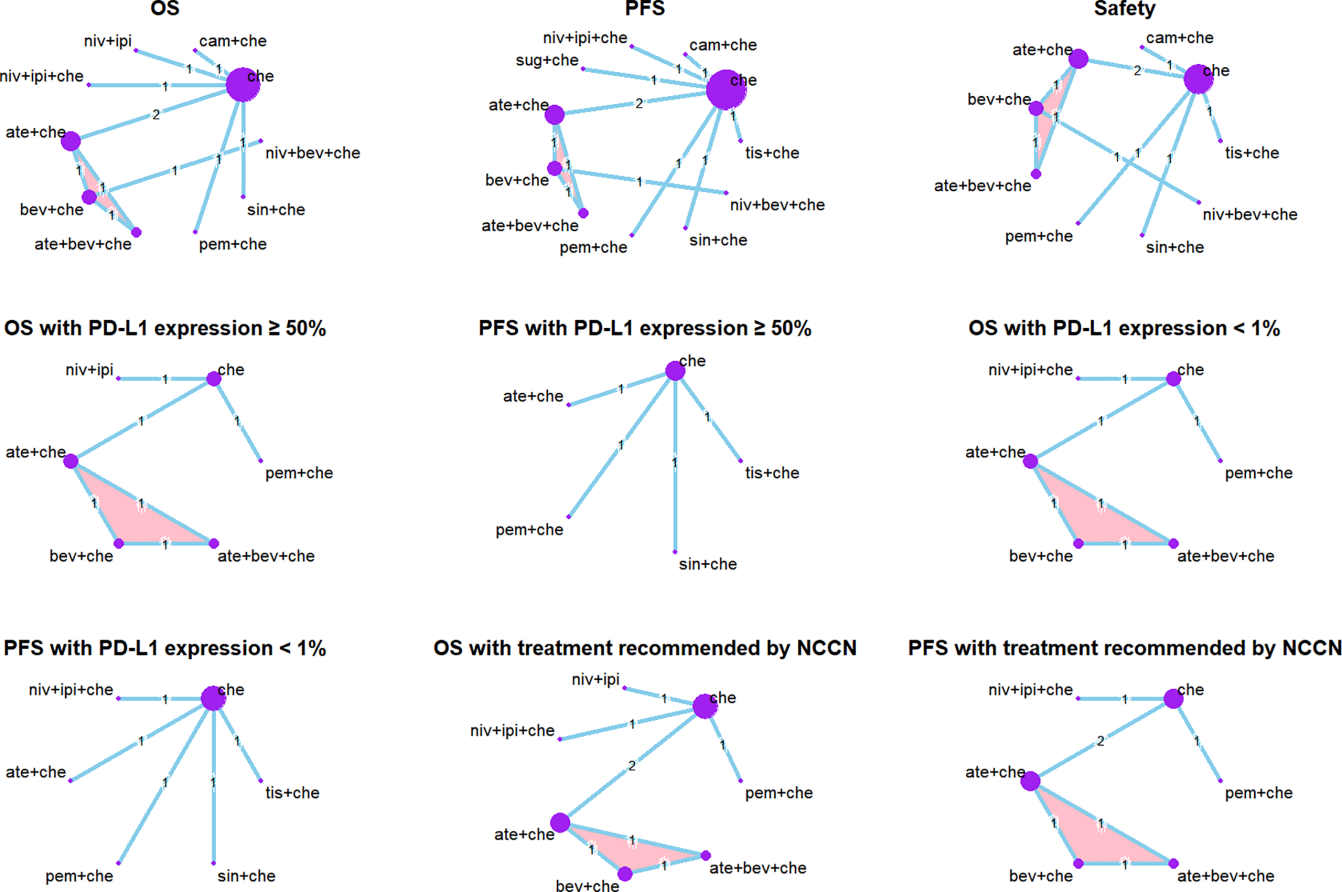
Network plots. Each circle represents an intervention as a node in the network. The size of the circle is proportional to the number of randomized controlled trials. Multi-arm study is highlighted. Safety: the network plots of analysis of AEs of any grade and grade ≥ 3 AEs are the same.

We identified a total of 700 records from the databases: 526 articles, 32 trial restriction records, and 142 abstracts. After removing duplicates, 580 records were left for abstract screening, 35 studies were considered eligible for full-text review, and 17 studies met our eligibility criteria (including 11 trials in total) (20, 21, 35–49). The PRISMA diagram is shown in **Figure 1**. Detailed information on all included trials is presented in **Table 1**. A total of 6,130 patients from the trials had received the following 12 treatments: chemotherapy (che), niv + ipi, sugemalimab plus chemotherapy (sug + che), ate + che, camrelizumab plus chemotherapy (cam + che), niv + ipi + che, ate + bev + che, bevacizumab plus chemotherapy (bev + che), nivolumab plus bevacizumab plus chemotherapy (niv + bev + che), pem + che, tislelizumab plus chemotherapy (tis + che), and sintilimab plus chemotherapy (sin + che). In the case of the Checkmate 227, Gemstone 302, and Checkmate 9LA trials, we included only patients with nsq-NSCLC. In the case of the IMpower-130 and IMpower-150 trials, we included only wild-type populationswere included. Note that the PFS curve of patients with nsq-NSCLC in the ate + che group versus bev + che group in the IMpower-150 trial could not be obtained; instead, we used the PFS curve of the entire intention-to-treat population. The network plots are depicted in **Figure 2**. The assessment of risk of bias is presented in **Supplementary Figure 3**. The Egger regression test was carried out to determine the publication bias, and *p*-values of 0.86 and 0.81 for PFS and OS, respectively, suggested the absence of publication bias in the included studies. The funnel plots are shown in **Supplementary Figure 4**.

**Table 1:** Baseline characteristics of included studies.

Study	Source (year)	Registered ID	Sample size*	Stage	Age*	Sex*	Included sample size*	Ethnicity (%)	Intervention arms	Control arms	Reported outcomes	Included subgroups^†^
Checkmate 227	JTO (2021)	NCT02477826	583/583	IV	64/64	778/388	419/419	White (63.3); Asian (19.2); other (17.2)	Arm 1: nivolumab 3 mg/kg Q2W + ipilimumab 1 mg/kg Q6W	Chemotherapy (carboplatin AUC 6 or cisplatin 75 mg/m^2^ + pemetrexed 500 mg/m^2^ Q3W)	OS, AEs of any grade, grade ≥ 3 AEs	OS: PD-L1 ≥ 50%
									Arm 2: nivolumab 240 mg Q2W			
Gemstone 302	Lancet Oncol (2022)	NCT03789604	320/159	IV	62/64	383/96	129/63	Asian (100.0)	Sugemalimab 1,200 mg Q3W + chemotherapy	Chemotherapy (carboplatin AUC 5 + pemetrexed 500 mg/m^2^ Q3W)	PFS, any grade AEs, grade ≥ 3 AEs	/
Impower 132	JTO (2020)	NCT02657434	292/286	IV	64/63	384/194	292/286	White (68.5); Asian (23.5)	Atezolizumab 1,200 mg Q3W + chemotherapy	Chemotherapy (carboplatin AUC 6 or cisplatin 75 mg/m^2^ + pemetrexed 500 mg/m^2^ Q3W)	PFS, OS, AEs of any grade, grade ≥ 3 AEs	OS, PFS: PD-L1 ≥ 50%, PD-L1< 1%
CameL	Lancet Respir Med (2021)	NCT03134872	205/207	IIIB–IV	59/61	295/117	205/207	Asian (100.0)	Camrelizumab 200 mg Q3W + chemotherapy	Chemotherapy (carboplatin AUC 5 + pemetrexed 500 mg/m^2^ Q3W)	PFS, OS, AEs of any grade, grade ≥ 3 AEs	/
Checkmate 9LA	ESMO Open (2021)	NCT03215706	361/358	IV	65/65	215/504	246/246	White (89.2); Asian (8.3); Black (1.4); other (1.1)	Nivolumab 350 mg Q3W + ipilimumab 1 mg/kg Q6W + chemotherapy	Chemotherapy (carboplatin AUC 6 or cisplatin 75 mg/m^2^ + pemetrexed 500 mg/m^2^ Q3W)	PFS, OS, AEs of any grade, grade ≥ 3 AEs	PFS: PD-L1< 1%
Impower 150	JTO (2021)	NCT02366143	356/350/336	IV	63/62/63	625/417	356/350/336	White (82.1); Asian (12.5); Black (1.5); other (3.9)	Arm 1: atezolizumab 1,200 mg Q3W + bevacizumab 15 mg/kg Q3W + chemotherapy	Bevacizumab 15 mg/kg Q3W + chemotherapy (carboplatin AUC 6 + paclitaxel 200 mg/m^2^ Q3W)	PFS, OS, AEs of any grade, grade ≥ 3 AEs	OS: PD-L1 ≥ 50%, PD-L1< 1%
									Arm 2: atezolizumab 1,200 mg Q3W + chemotherapy			
TASUKI 52	Ann Oncol (2021)	NCT03117049	275/275	IIIB–IV	66/66	411/139	275/275	Asian (100.0)	Nivolumab 360 mg Q3W + bevacizumab 15 mg/kg Q3W + chemotherapy	Bevacizumab 15 mg/kg Q3W + chemotherapy (carboplatin AUC 6 + paclitaxel 200 mg/m^2^ Q3W)	PFS, OS, AEs of any grade, grade ≥ 3 AEs	/
Keynote 189	Ann Oncol (2021)	NCT02578680	410/206	IV	65/63.5	363/253	410/206	White (86.2); Asian (1.6); other (12.2)	Pembrolizumab 200 mg Q3W + chemotherapy	Chemotherapy (carboplatin AUC 5 or cisplatin 75 mg/m^2^ + pemetrexed 500 mg/m^2^ Q3W)	PFS, OS, AEs of any grade, grade ≥ 3 AEs	OS: PD-L1 ≥ 50%, PD-L1< 1%
Rationale 304	JTO (2021)	NCT03663205	223/111	IIIB–IV	60/61	247/87	223/111	Asian (100.0)	Tislelizumab 200 mg Q3W + chemotherapy	Chemotherapy (carboplatin AUC 5 or cisplatin 75 mg/m^2^ + pemetrexed 500 mg/m^2^ Q3W)	PFS, AEs of any grade, grade ≥ 3 AEs	PFS: PD-L1 ≥ 50%, PD_L1< 1%
Orient 11	Ann Oncol (2022)	NCT03607539	266/131	IIIB–IV	61/61	303/94	266/131	Asian (100.0)	Sintilimab 200 mg Q3W + Chemotherapy	Chemotherapy (carboplatin AUC 5 or cisplatin 75 mg/m^2^ + pemetrexed 500 mg/m2 Q3W)	PFS, OS, AEs of any grade, grade ≥ 3 AEs	PFS: PD-L1 ≥ 50%, PD-L1< 1%
Impower 130	Lancet Oncol (2019)	NCT02367781	451/228	IV	Age:64/65	Sex:400/279	451/228	White (90.1); Asian (2.3); Black (3.8); other (3.8)	Atezolizumab 1,200 mg Q3W + Chemotherapy	Chemotherapy(carboplatin AUC 6 Q3W + nab-paclitaxel 100 mg/m^2^ QW)	PFS, OS, AEs of any grade, grade ≥ 3 AEs	/

* Sample size, age, sex, and included sample are in the order of intervention arm/control arm. Sample size is the number of people included in the original trial. Included sample size is the number of included people in this NMA.

^†^The included subgroups column shows PFS/OS data by subgroup of PD-L1 expression (PD-L1 expression ≥ 50% and/or PD-L1 expression< 1%).

Q2W, every 2 weeks; Q3W, every 3 weeks; Q6W, every 6 weeks.

### Results for overall survival

**Figure 3:**
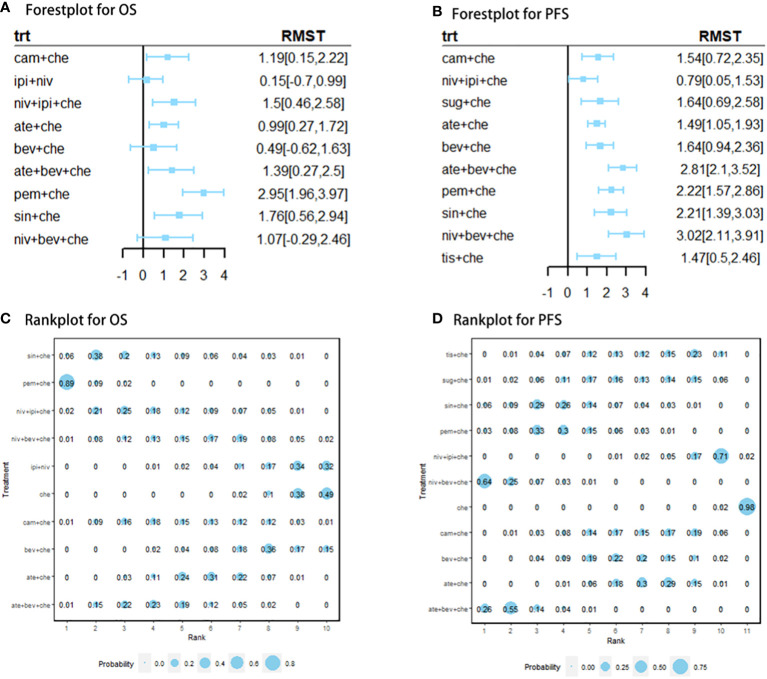
**(A)** forest plot of RMST for OS; **(B)** forest plot of RMST for PFS; **(C)** rank plot for OS; **(D)** rank plot for PFS. Forest plot of restricted mean survival time (RMST) and rank plot for overall survival (OS) and progression-free survival (PFS). In the rank plot, the size of each point is proportional to the probability (i.e., the probability of being the best treatment in terms of survival benefits at 12 or 18 months).

**Figure 4:**
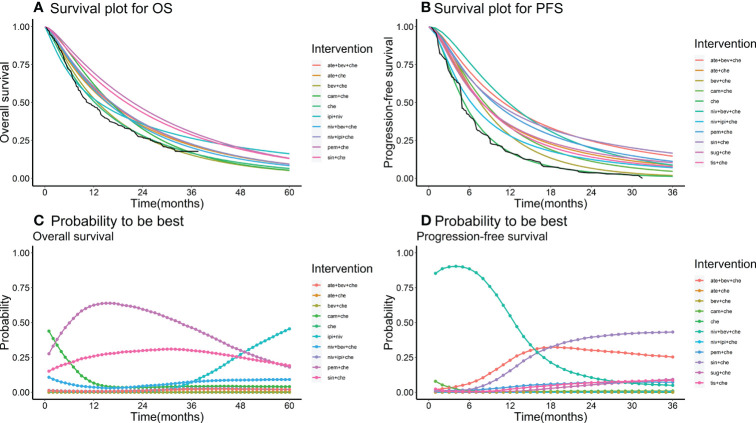
**(A)** survival plot for OS; **(B)** survival plot for PFS; **(C)** rank plot for OS; **(D)** rank plot for PFS. Survival plot and rank plot for overall survival (OS) and progression-free survival (PFS). The black line in the survival plots is the Kaplan–Meier (KM) curve of the reference chemotherapy. Probability is the probability of being the best treatment in terms of survival benefits in each month from month 36 to month 60.

Nine studies of 10 treatments were included in the NMA of OS (**Figure 2**). Patients who received ICI combination therapies were more likely to obtain OS benefit than those who received only chemotherapy. In terms of OS at 18 months (**Figures 3A, C**), compared with chemotherapy, pem + che yielded the best OS benefit (RMST 2.95, 95% CI 1.96 to 3.97), with a probability of ranking first among all ICI combination therapies of 89%. Sin + che (RMST 1.76, 95% CI 0.56 to 2.9) and ate + bev + che (RMST 1.39, 95% CI 0.27 to 2.5), were found to rank second and third, respectively, for OS, with a probability of ranking first of 38% and 22%, respectively. In terms of OS at 60 months (**Figure 4A** and **Table 2**), pem + che still yielded the best OS benefit, providing an life-years gain over a 5-year period of 2.18 years. Sin + che was found to be comparable to pem + che (with a life-years gain over a 5-year period of 2.09 months). When the results were broken down by time period, cam + che ranked first in OS benefits in the first 2 months, with pem + che having the highest probability of ranking first for the following 47 months, and niv + ipi being the combination most likely to rank first from month 49 to month 60 (**Figure 4C**).

**Table 2:** Life-years gained of OS and PFS.

Intervention	LE_5y (years)	Intervention	LE_3y (years)
che	1.51 years	che	0.58 years
cam + che	1.68 years	cam + che	0.89 years
ipi + niv	1.83 years	niv + ipi + che	0.81years
niv + ipi + che	1.82 years	sug + che	0.97years
ate + che	1.76 years	ate + che	0.95 years
bev + che	1.5 years	bev + che	0.77 years
ate + bev + che	1.8 years	ate + bev + che	1.22 years
pem + che	2.18 years	pem + che	1.1 years
sin + che	2.09 years	sin + che	1.19 years
niv + bev + che	1.73 years	niv + bev + che	1.18 years
		tis + che	0.92 years

LE_3Y, life -years gained of PFS at 3 years; LE_5Y, life -years gained of OS at 5 years.

### Results for progression-free-survival

Ten studies considering 11 treatments were included in the NMA of PFS (**Figure 2B**). All ICI combination therapies were associated with greater PFS benefits than chemotherapy alone. The greatest PFS benefit at 12 months compared with chemotherapy (**Figures 3B, D**) was achieved with niv + bev + che (RMST 3.02, 95% CI 2.11 to 3.91), with this combination therapy having a probability of ranking first of 64%, followed by, in order, ate + bev + che (RMST 2.81, 95% CI 2.1 to 3.52; probability of ranking first 55%), pem + che (RMST 2.22, 95% CI 1.57 to 2.86; probability of ranking first 33%), and sin + che (RMST 2.21, 95% CI 1.39 to 3.03, probability of ranking first 26%). In the case of PFS at 36 months (**Figure 4B** and **Table 2**), ate + bev + che yielded the best PFS benefit, in terms of life-years gained over a 3-year period, of 1.22 years. Niv + bev + che and sin + che were found to be comparable to ate + bev + che, with life-years gained over a 3-year period of 1.18 years and 1.19 years, respectively. Niv + bev + che ranked first in PFS benefits for the first 15 months, while ate + bev + che ranked first from month 16 to month 17, and sin + che was the most likely to rank first for the remainder of the 36-month period (**Figure 4D**).

### Safety

Safety was compared by analyzing the occurrence of AEs of any grade and of grade ≥ 3 AEs. This NMA included eight studies of nine treatments (**Figure 2**). Point estimates of ORs revealed that most combination therapies, except sin + che and niv + bev + che, were associated with more AEs of any grade than chemotherapy alone (see **Supplementary Figure 5**). ORs for sin + che equaled 0 because all patients in the chemotherapy group in the Orient trial experiences AEs. Combination therapies in which anti-angiogenesis drugs were administered simultaneously with ICIs showed better safety performance than those that did not include anti-angiogenesis drugs (ate + bev + che: OR 2.83, 95% CI 0.66 to 12.94; niv + bev + che: OR 0.93, 95% CI 0.02 to 15.03). The pem + che and sin + che combination was associated with relatively fewer grade ≥ 3 AEs than the other combination therapies (see **Supplementary Figure 5**); however, no statistically significant differences between these two treatments and other combination therapies were found. In addition, some combination ICI treatments were associated with more grade ≥ 3 AEs than chemotherapy, including cam + che (OR 2.46, 95% CI 1.66 to 3.72), ate + che (OR 1.67, 95% CI 1.29 to 2.15), bev + che (OR 1.81, 95% CI 1.23 to 2.66), and ate + bev + che (OR 2.45, 95% CI 1.65 to 3.63).

### Subgroup analysis

Network plots of subgroup analysis are shown in **Figure 2**.

**Figure 5:**
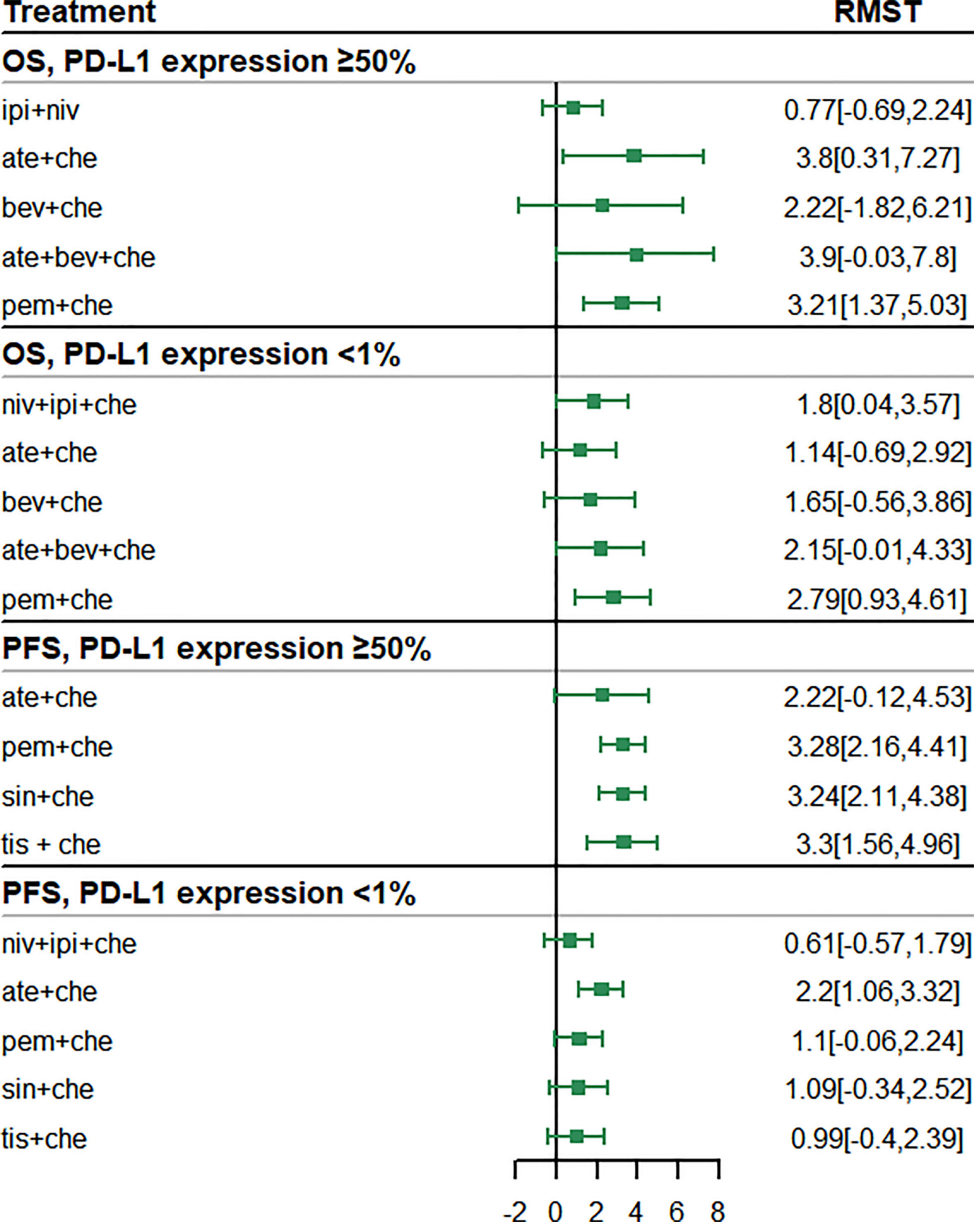
Forest plots of restricted mean survival time (RMST) in the subgroup analysis of programmed cell death ligand 1 (PD-L1) expression.

The subgroup analysis of patients with PD-L1 expression ≥ 50% included five studies of six treatments in the PFS comparison and four studies of six treatments in the OS comparison. Compared with chemotherapy, both ate + che (RMST 3.8, 95% CI 0.31 to 7.27) and pem + che (RMST 3.21, 95% CI 1.37 to 5.03) were associated with significantly higher OS at 18 months (**Figure 5**). When extrapolated to 60 months, niv + ipi showed the best OS benefits, with a 5-year life-years gain of 2.69 years, followed by pem + che, with a 5-year life-years gain of 2.59 years (**Table 3**).

**Table 3:** Life-years gained by patients with different levels of PD-L1 expression.

Patients with PD-L1 expression ≥ 50%				Patients with PD-L1 expression < 1%			
Intervention	LE_5y (years)	Intervention	LE_3y (years)	Intervention	LE_5y (years)	Intervention	LE_3y (years)
che	2.11 years	che	0.55 years	che	1.05 years	che	0.55 years
ipi + niv	2.69 years	ate + che	1.18 years	niv + ipi + che	1.32 years	niv + ipi + che	0.84 years
ate + che	2.4 years	pem + che	1.37 years	ate + che	1.51 years	ate + che	1.39 years
bev + che	2.09 years	sin + che	1.52 years	bev + che	1.5 years	pem + che	0.8 years
ate + bev + che	2.5 years	tis + che	1.42 years	ate + bev + che	1.62 years	sin + che	0.88 years
pem + che	2.59 years			pem + che	1.79 years	tis + che	0.83 years

LE_3Y, increase in PFS (life-years) at 3 years; LE_5Y, increase in OS (life-years) iat5 years.

In the case of PFS, the greatest benefit at 12 months, compared with chemotherapy, was achieved with tis + che (RMST 3.3, 95% CI 1.56 to 4.96), followed by pem + che (RMST 3.28, 95% CI 2.16 to 4.41) and sin + che (RMST 3.24, 95% CI 2.11 to 4.38) (**Figure 5**). When extrapolated to 36 months, sin + che showed the best PFS benefits, with a life-years gain over a 3-year period of 1.52 years (**Table 3**). All ICI combination therapies achieved greater PFS benefits than chemotherapy. Survival plots and rank plots for long-term effects are shown in **Supplementary Figures 6** and **7**. Rank plots for RMST are shown in **Supplementary Figure 8**.

The subgroup analysis of patients with PD-L1 expression< 1% included four studies of five treatments in the PFS comparison and four studies of six treatments in the OS comparison. Regarding OS, pem + che (RMST 2.79, 95% CI 0.93 to 4.61) and niv + ipi + che (RMST 1.8, 95% CI 0.04 to 3.57) were associated with significantly prolonged survival benefits at 18 months compared with chemotherapy (**Figure 5**). When extrapolated to 60 months, pem + che showed the best OS benefits, with a 5-year life-years gain of 1.79 years, followed by ate + bev + che, with a life-years gain of 1.62 years (**Table 3**).

Compared with chemotherapy, only ate + che achieved significantly better PFS at 12 months (RMST 2.2, 95% CI 1.06 to 3.32) (**Figure 5**). When extrapolated to 36 months, ate + che still showed the best PFS benefits, with a 3-year life-years gain of 1.39 years (**Table 3**). Survival plots and rank plots for long-term effects are shown in **Supplementary Figures 6** and **7**.

For those ICI combination therapies recommended by the NCCN, the results of six studies involving a total of seven treatments were compared. The results of the RMST analysis were consistent with our base-case analysis (see **Supplementary Figure 9**). For long-term effects, the life-years gain was estimated to be slightly lower than in the base-case analysis but the ranking order was the same (see **Supplementary Figure 10** and **Supplementary Table 2**).

### Convergence and heterogeneity assessment

We used the history feature to estimate the convergence of NMA models. The Brooks–Gelman–Rubin method revealed that the three Markov chains were stable and replicable of the inferential iterations in all models. The results of the pairwise meta-analysis are shown in **Supplementary Figure 11**. The findings of the *Q*-test, the *I* ^2^ statistic, and the forest plots all revealed that there was low heterogeneity across the specific arm.

## Discussion

In this study, we used RMST to compare the short-term effects of different ICI combination therapies, and the Royston–Parmar model to extrapolate and synthesize the long-term effects. Our principal findings can be summarized as follows:

1. ICI combination therapies provided PFS and OS benefits superior to chemotherapy alone both in the short term and in the long term.
2. For patients with advanced nsq-NSCLC without PD-L1 selection, pem + che exhibited the best OS benefit at both 18 and 60 months, and niv + bev + che was found to present the best PFS benefits at 12 months, whereas ate + bev + che showed the best PFS benefits at 36 months.
3. For patients with PD-L1 expression ≥ 50%, ate + che and niv + ipi presented the best OS benefits at 18 and 60 months, respectively. Tis + che and sin + che exhibited the best PFS benefits at 12 and 36 months, respectively.
4. For patients with PD-L1 expression< 1%, pem + che showed the best OS benefits at both 18 and 60 months. Only ate + che significantly improved PFS benefits at both 12 months and 36 months.
5. Although ICI combination therapies improve survival, they may increase the risk of AEs. Combinations of ICIs plus anti-angiogenesis drugs may improve safety, but we found no statistical differences in the prevalence of AEs of any grade or of grade ≥ 3 AEs among ICI combination therapies.

In recent years, several systematic reviews and NMAs have targeted first-line immunotherapies for the treatment of patients with advanced NSCLC but without alterations in epidermal growth factor receptor (EGFR) or anaplastic lymphoma kinase (ALK). However, few studies have directly focused on the non-squamous histologic type. Frederickson et al. (50) found that pembrolizumab plus pemetrexed plus platinum was likely to be the most efficacious first-line regimen for metastatic nsq-NSCLC. Chai et al. (51) found that immunotherapy plus chemotherapy could prolong OS and PFS in nsq-NSCLC patients compared with bevacizumab plus chemotherapy.

Given differences in the clinical effects of different therapies, and the fact that EGFR or ALK testing was not mandatory for patients with NSCLC of squamous histologic type (4, 52), a popular practice in the past was to conduct a subgroup analysis based on different histologic types. For example, subgroup analyses carried out by Liu et al. (16) and Sheng et al. (53) found that the optimal combination ICIs therapies were different in patients with squamous NSCLC and those with nsq-NSCLC (16, 53). Note that previous NMAs were largely based on the PH assumption, and, therefore, were unable to estimate the long-term effects of ICIs. Herbst et al. (54) and Vickers et al. (55) carried out NMAs with fractional polynomial models based on the assumption of non-PH. However, their studies did not include first-line treatment of nsq-NSCLC patients. Therefore, our study is the first to evaluate both the short-term and long-term effects of first-line ICI combination therapies for patients with nsq-NSCLC without EGFR or ALK alterations. Our principal findings were similar to the results of Liu et al.’s *(* 16) study, which was conducted with HRs under the assumption of PH. However, our study reported RMST and life-years gained for each specific treatment., Such information, being directly related to clinical benefit, is likely to be more readily understood by clinicians and patients and to be considered more relevant when choosing the optimal therapy.

PD-L1 expression has become a popular biomarker among clinicians, who use it to tailor treatment regimens (56, 57). Higher PD-L1 expression is associated with poor prognosis (58–60). With the emergence of immunotherapy, studies have shown that, in general, the higher the expression of PD-L1, the greater the benefit of immunotherapy (61).

Several published NMAs have considered populations with different levels of PD-L1 expression l as potential subgroups (16, 53, 57, 62). For example, Liu et al. (16) reported that the greatest OS was achieved by niv + ipi + che in patients with PD-L1 expression< 1% and by pem + che in patients with PD-L1 expression ≥ 1%. Sheng et al. (53) and Wang et al. (62) found that, among nsq-NSCLC patients with high PD-L1 expression, ICI combination therapies were associated with significantly greater OS and PFS benefits than ICI-free therapies, but significant PFS benefits only when compared with ICIs alone therapy (53, 62). However, Passiglia et al. (57) found that ICI combinations had limited effects in patients with high PD-L1 expression, but might be a suitable option for the subgroup who are PD-L1 negative.

In contrast to these studies, our study focused on the population with nsq-NSCLC. We found that patients with PD-L1 expression ≥ 50% obtained greater survival benefits than patients negative for PD-L1 expression. In our study, several ICI combination therapies significantly improved survival among patients with PD-L1 expression ≥ 50%, including niv + ipi (best OS benefits in the long term) and sin + che (best OS benefits in the short term). Notably, niv + ipi was the only ICI + ICI combined therapy included in this study. This indicates that ICI + ICI combinations could be of benefit in patients with high PD-L1 expression, but further studies are required to prove this hypothesis. For patients without PD-L1 expression, the only suitable option might be pembrolizumab (with prolonged OS benefits) and atezolizumab (with prolonged PFS benefits). Our findings could provide more evidence enabling clinicians to select the optimal therapy according to the individual patient characteristics. However, in contrast to previous studies, we did not carry out subgroup analyses comparing patients with PD-L1 expression > 1% and those with PD-L1 expression ≥ 1% but ≤ 49% owing to limitations of the data. It is noteworthy that the optimal treatments for subgroups with different levels of PD-L1 expression in our study are different from those reported by others (16, 53), which may reflect differences in the outcomes reported (HR, RMST, and life-years gained). Future real-world studies are needed to further help clinicians make appropriate choices.

Some specific ICI combination therapies, such as ICIs plus anti-angiogenesis drugs and ICIs plus other ICIs, showed significant clinical benefits, improving PFS in all patients and both PFS and OS in specific subgroups, which indicates that combination therapies have potential synergistic effects. Anti-angiogenesis drugs such as bevacizumab have been shown to improve survival in nsq-NSCLC patients when added to chemotherapy (63, 64). In combination with ICIs such as atezolizumab and nivolumab, by reversing vascular endothelial growth factor (VEGF)-mediated immunosuppression, bevacizumab can increase the effect of these ICIs (65). Niv + ipi, a combined ICI + ICI therapy (a combination of an anti-PD-1 drug and an anti-CTLA-4 drug), has been approved by the FDA for the treatment of several cancer types. This combination might aid in eliminating the tumor cells, i.e., ipilimumab supports the activation and proliferation of T cells, whereas nivolumab helps existing T cells to identify and target tumor cells (16, 66). Despite encouraging clinical benefits in terms of PFS in specific subgroups when compared with chemotherapy and ICIs plus chemotherapy, these combination therapies did not show OS benefits in our NMA when compared with ICIs plus chemotherapy. Our study suggests that further research into ICI + ICI therapies is necessary to develop treatments targeted at specific subgroup populations (e.g., those with a high of level PD-L1 expression).

To the best of our knowledge, this study is the first systematic review and NMA comparing both the short-term and the long-term effects of currently available ICI combination therapies for the treatment of patients with advanced nsq-NSCLC without EGFR or ALK alterations. We innovatively used RMST as a short-term outcome indicator. Compared with using HR as traditional outcome indicators, RMST could directly reflect the short-term survival benefits of patients receiving different ICI combination therapies and could also break through the limitation of PH assumptions.

Keynote 024 was the first study to reveal that pembrolizumab monotherapy could prolong OS in nsq-NSCLC patients to almost 5 years. Currently, follow-up in many trials of nsq-NSCLC does not reach year 5, and the average follow-up time is between 24 and 36 months. Some ICIs produced by Chinese manufacturers have been shown to be associated with PFS benefits (for example, CameL, Gemstone 302, and Rationale 304). However, OS benefits could not be exactly estimated because of the limited follow-up time. The approach used in our study could solve this data gap by using Royston–Parmar models for NMA modeling to extrapolate the long-term effects. The results of our study will enable clinicians and patients to determine the long-term survival benefits of newly emerged therapies and to choose the optimal therapy. The results of our subgroup analyses could also help physicians to tailor the treatment regimen for patients with specific PD-L1 expression levels. Our robust methodology and synthesis mean that our results are reliable and relevant to clinical practice.

However, our study also has several limitations. First, although we projected the short-term effect to a longer time horizon, OS data are still being followed up in the CameL, Rationale 304, Orient 11, and Gemstone302 trials, which may lead to heterogeneity and risk of bias. However, subgroup analysis based on therapies recommended by NCCN found no significant risk of bias when these studies were included. Second, because KM curves were needed, we were unable to carry out some key subgroup analyses, including analyses according to the level of PD-L1 expression (> 1% compared with ≥ 1% but ≤ 49%) or sex, ethnicity, or region. Furthermore, the only predictive biomarker considered in this study was PD-L1 expression level, which could provide only limited recommendations for clinicians. Well-designed clinical trials that provide more comprehensive data are needed in the future. Third, our NMA assumed that all patients received the same type of chemotherapy, when in fact patients received one of two chemotherapeutic regimens: pemetrexed based and pemetrexed free (paclitaxel/nab-paclitaxel). Pemetrexed is more effective than other third-generation chemotherapies in patients with nsq-NSCLC. However, it is still hard to tell which chemotherapeutic strategy is more effective when combined with ICIs. Fourth, although rank plot models could fit and extrapolate survival data better, overfitting could be observed when survival data were immature. This indicates that a more comprehensive methodology for extrapolating survival data, if and when such a methodology becomes available, should be considered. Fifth, owing to the crossover design of the included trial, the relative OS benefits in the intervention group may be underestimated, and so the synthesized results should be considered carefully. Finally, although the conclusions in this study could inform physicians’ choices of immunotherapies for patients with nsq-NSCLC, further validation is needed before these conclusions can be implemented in clinical practice.

## Conclusions

Our study revealed that ICI combination therapies are associated with better survival benefits than chemotherapy alone. ICIs plus chemotherapy performed well in almost all comparisons: ICIs plus anti-angiogenesis drugs showed potentially better PFS benefits than other combination therapies, and ICI + ICI combinations exhibited better survival benefits than other combination therapies in patients with PD-L1 expression ≥ 50%. Our results could provide more understandable evidence to clinicians and patients to choose the optimal therapy. This innovative study framework of non-PH assumption-based NMA could also provide a reference for other researchers. Furthermore, long-term follow-up data and well-designed head-to-head trials are needed for patients with nsq-NSCLC as well as their subgroups.

## Data availability statement

The original contributions presented in the study are included in the article/**Supplementary Material**. Further inquiries can be directed to the corresponding author. R codes for this study are available from GitHub (https://github.com/TaihangShao/nsq_scope_NMA).

## Author contributions

Conceptualization: TS and WT. Methodology: MZ and TS. Analysis and visualization: TS and LL. Writing – original draft preparation: TS and MZ. Writing – review and editing: LL and WT. Funding acquisition: WT. Supervision: WT. All authors read and approved the final manuscript.

## Funding

General Program of National Natural Science Foundation of China (72174207).

## Conflict of interest

The authors declare that the research was conducted in the absence of any commercial or financial relationships that could be construed as a potential conflict of interest.

## Publisher’s note

All claims expressed in this article are solely those of the authors and do not necessarily represent those of their affiliated organizations, or those of the publisher, the editors and the reviewers. Any product that may be evaluated in this article, or claim that may be made by its manufacturer, is not guaranteed or endorsed by the publisher.
