# Supplementary material for: A systematic review and network meta-analysis of first-line immune checkpoint inhibitor combination therapies in patients with advanced non-squamous non-small cell lung cancer
Source: Front Immunol. 2022 Oct 26;13:948597. doi: 10.3389/fimmu.2022.948597 (PMC9645411; doi:10.3389/fimmu.2022.948597)
Supplement: Supplementary file 1 [file DataSheet_1.docx]

**Supplementary Material**

**Content**

[Supplementary Table 1 2](#_Toc27311)

[Supplementary Table 2 3](#_Toc10354)

[Supplementary Figure 1 4](#_Toc12856)

[Supplementary Figure 2 7](#_Toc7658)

[Supplementary Figure 3 10](#_Toc2361)

[Supplementary Figure 4 11](#_Toc8141)

[Supplementary Figure 5 12](#_Toc26495)

[Supplementary Figure 6 13](#_Toc28492)

[Supplementary Figure 7 14](#_Toc30631)

[Supplementary Figure 8 15](#_Toc2558)

[Supplementary Figure 9 16](#_Toc28852)

[Supplementary Figure 10 17](#_Toc17299)

[Supplementary Figure 11 18](#_Toc21860)

#### Supplementary Table 1

**Search strategies**

| No | Term |
| --- | --- |
| PubMed | |
| #1 | (clinical trial[Title/Abstract]) OR (trial[Title/Abstract]) OR (randomized controlled trial[Title/Abstract]) OR (phase[Title/Abstract]) |
| #2 | (non-small cell lung cancer[Title]) OR (non-small-cell lung cancer[Title]) OR (non small-cell lung cancer[Title]) OR (non small cell lung carcinoma[Title]) OR (NSCLC[Title]) |
| #3 | (immune checkpoint inhibitor[Title]) OR (PD-1[Title]) OR (PD-L1[Title]) OR (CTLA-4[Title]) OR (pembrolizumab[Title]) OR (atezolizumab[Title]) OR (nivolumab[Title]) OR (ipilimumab[Title]) OR (durvalumab[Title]) OR (tremelimumab[Title]) OR (camrelizumab[Title]) OR (tislelizumab[Title]) OR (sintilimab[Title]) OR (Toripalimab[Title]) OR (Cemiplimab[Title]) OR (Sugemalimab[Title]) |
| Medline | |
| #1 | TS=('clinical trial' OR 'trial' OR 'randomized controlled trial' OR 'phase') |
| #2 | TI=('non-small cell lung cancer' OR 'non-small-cell lung cancer' OR 'non small-cell lung cancer' OR 'non small cell lung carcinoma' OR 'NSCLC') |
| #3 | TI=('immune checkpoint inhibitor' OR 'PD-1' OR 'PD-L1' OR 'CTLA-4' OR 'pembrolizumab' OR 'atezolizumab' OR 'nivolumab' OR 'ipilimumab' OR 'durvalumab' OR 'tremelimumab' OR 'camrelizumab' OR 'tislelizumab' OR 'sintilimab' OR 'Toripalimab' OR 'Cemiplimab' OR 'Sugemalimab') |
| Embase | |
| #1 | clinical trial':ti,ab,kw OR 'trial':ti,ab,kw OR 'randomized controlled trial':ti,ab,kw OR 'phase':ti,ab,kw |
| #2 | non-small cell lung cancer':ti OR 'non-small-cell lung cancer':ti OR 'non small-cell lung cancer':ti OR 'non small cell lung carcinoma':ti OR 'NSCLC':ti |
| #3 | immune checkpoint inhibitor':ti OR 'PD-1':ti OR 'PD-L1':ti OR 'CTLA-4':ti OR 'pembrolizumab':ti OR 'atezolizumab':ti OR 'nivolumab':ti OR 'ipilimumab':ti OR 'durvalumab':ti OR 'tremelimumab':ti OR 'camrelizumab':ti OR 'tislelizumab':ti OR 'sintilimab':ti OR 'Toripalimab':ti OR 'Cemiplimab':ti OR 'Sugemalimab':ti |
| #4 | #1 AND #2 AND #3 |
| #5 | #4 AND ([controlled clinical trial]/lim OR [randomized controlled trial]/lim) AND ([article]/lim OR [article in press]/lim OR [conference abstract]/lim OR [conference paper]/lim) |

#### Supplementary Table 2

| **Life-years gained of patients in subgroup analysis of NCCN recommended treatments** | | | |
| --- | --- | --- | --- |
| Intervention | LE_5y | Intervention | LE_3y |
| che | 18.04 | che | 6.91 |
| ipi+niv | 21.94 | niv+ipi+che | 9.69 |
| niv+ipi+che | 21.85 | ate+che | 11.37 |
| ate+che | 21.03 | bev+che | 9.30 |
| bev+che | 17.97 | ate+bev+che | 14.63 |
| ate+bev+che | 21.54 | pem+che | 13.18 |
| Che: chemotherapy; niv+ipi: nivolumab plus ipilimumab; ate+che: atezolizumab plus chemotherapy; niv+ipi+che: nivolumab plus ipilimumab plus chemotherapy; ate+bev+che: atezolizumab plus bevacizumab plus chemotherapy; bev+che: bevacizumab plus chemotherapy; pem+che: pembrolizumab plus chemotherapy; OS: overall survival; PFS: progression-free survival. LE_5Y: life-years gained of OS in 5 years; LE_3Y: life-years gained of PFS in 3 years. LE_5Y: life-years gained of OS in 5 years; LE_3Y: life-years gained of PFS in 3 years. | | | |

#### Supplementary Figure 1

| **Log Cumulative Hazard Plot** | | |
| --- | --- | --- |
| **OS** | | |
| 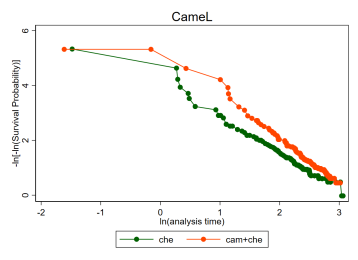 | 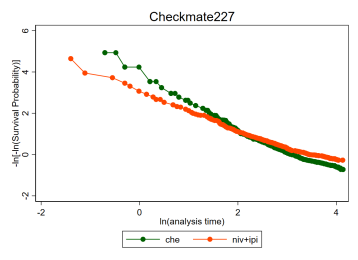 | 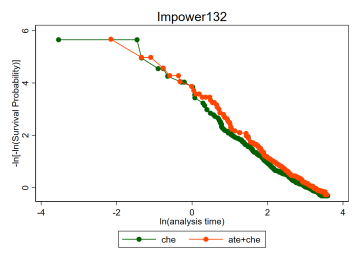 |
| 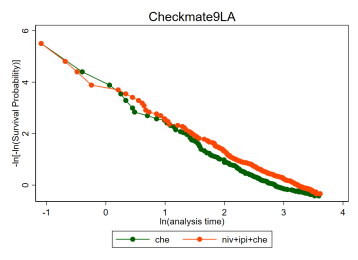 | 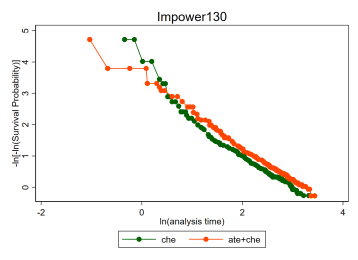 | 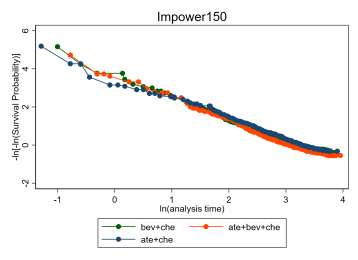 |
| 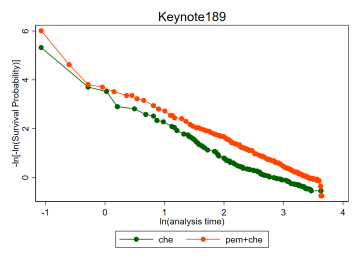 | 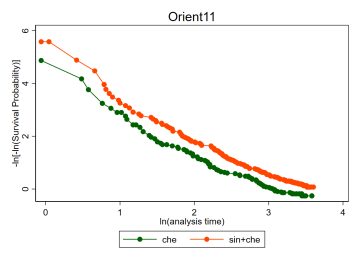 | 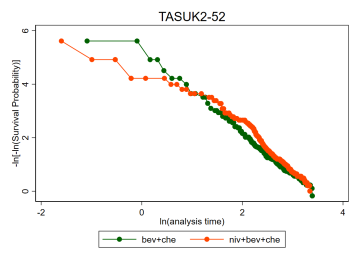 |
| **PFS** | | |
| 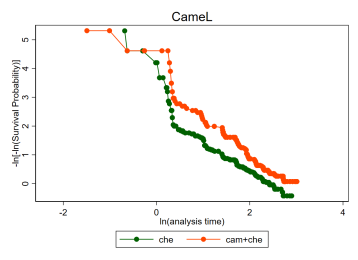 | 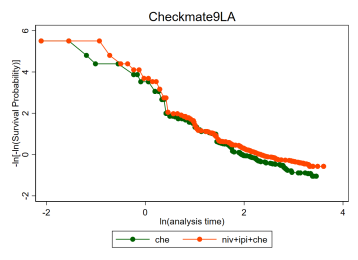 | 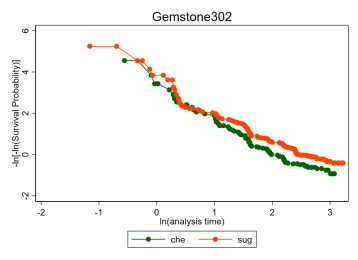 |
| 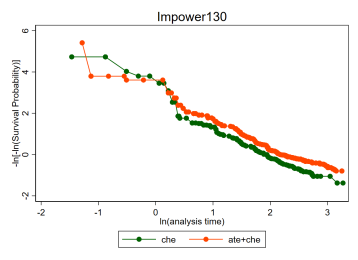 | 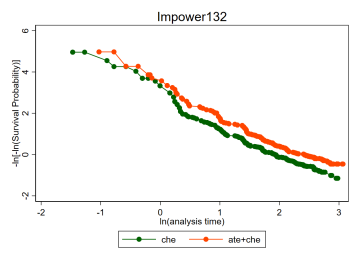 | 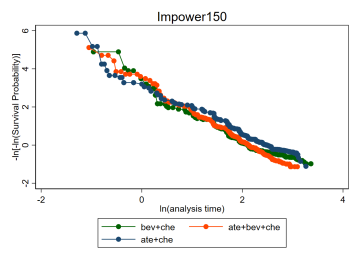 |
| 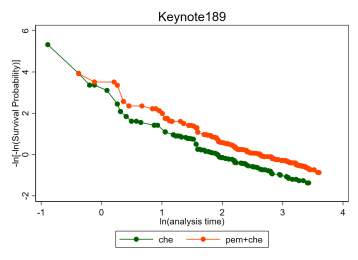 | 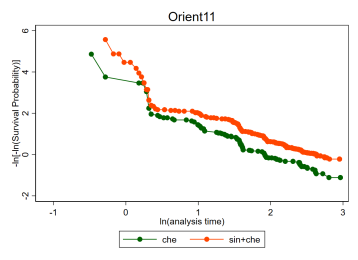 | 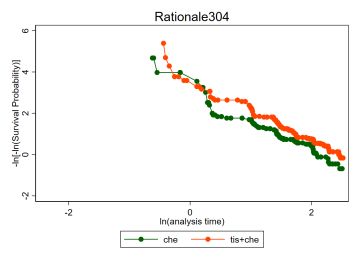 |
| 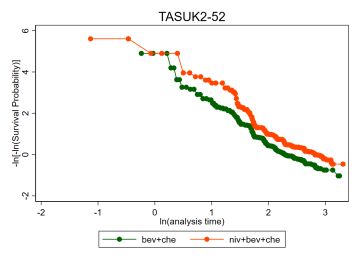 |  |  |
| **OS, PD-L1 expression ≥ 50%** | | |
| 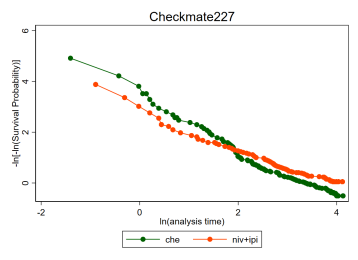 | 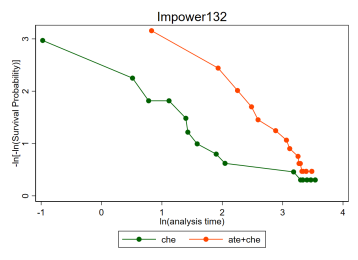 | 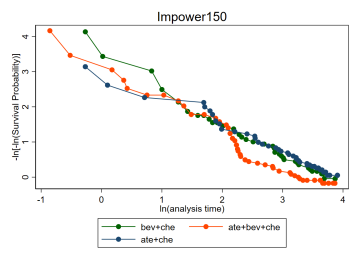 |
| 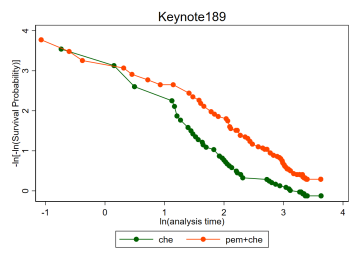 |  |  |
| **OS, PD-L1 expression < 1%** | | |
| 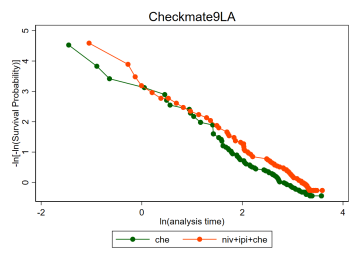 | 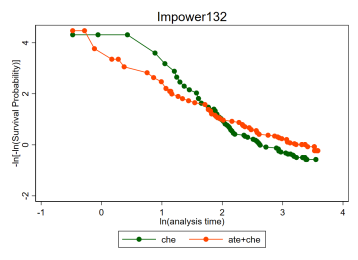 | 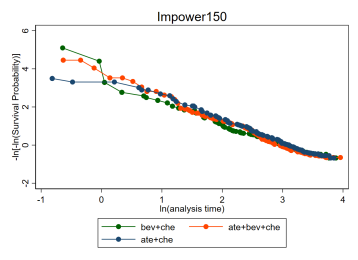 |
| 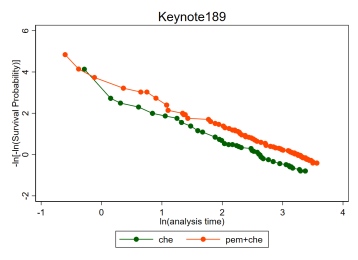 |  |  |
| **PFS, PD-L1 expression ≥ 50%** | | |
| 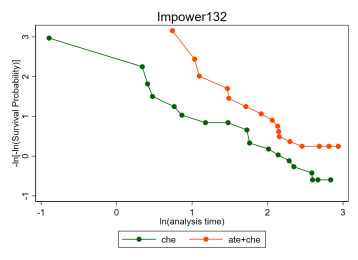 | 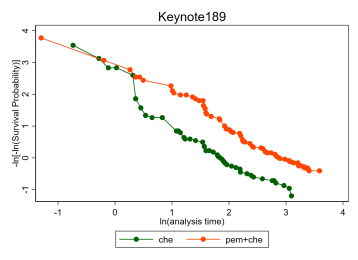 | 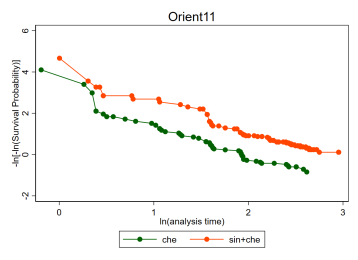 |
| 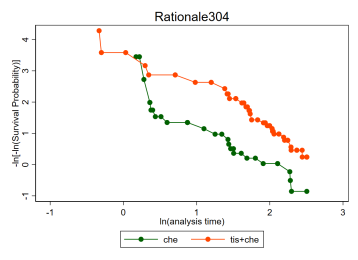 |  |  |
| **PFS, PD-L1 expression < 1%** | | |
| 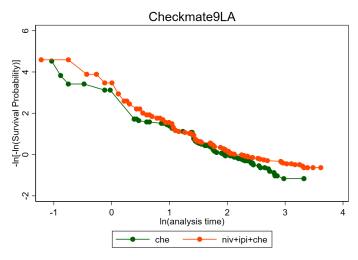 | 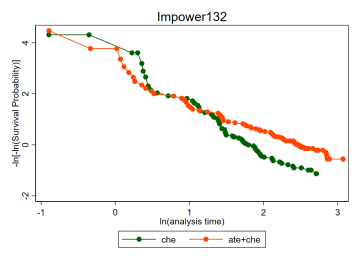 | 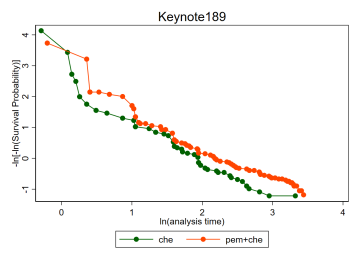 |
| 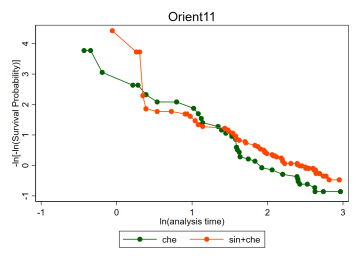 | 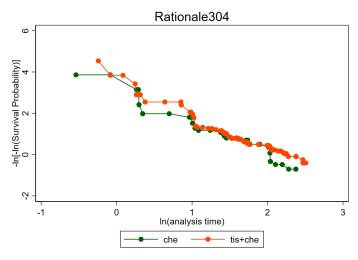 |  |

#### Supplementary Figure 2

| **Schoenfeld residual test Plot** | | |
| --- | --- | --- |
| **OS** | | |
| 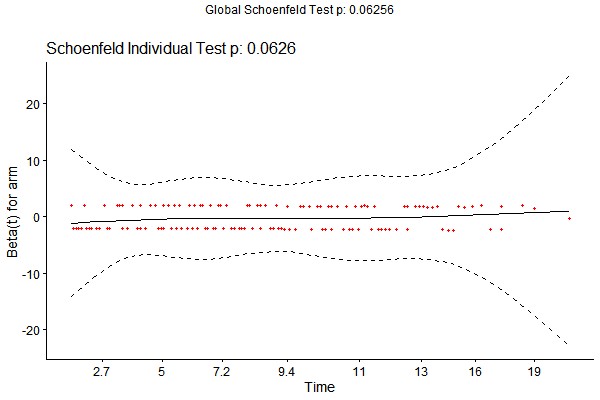 | 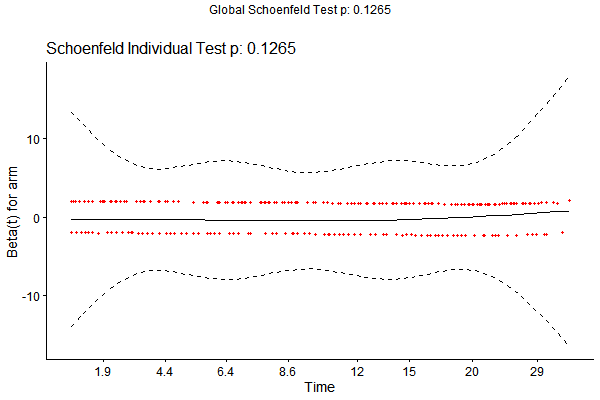 | 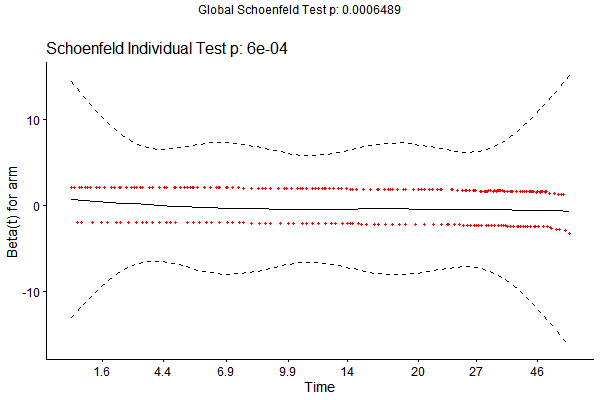 |
| 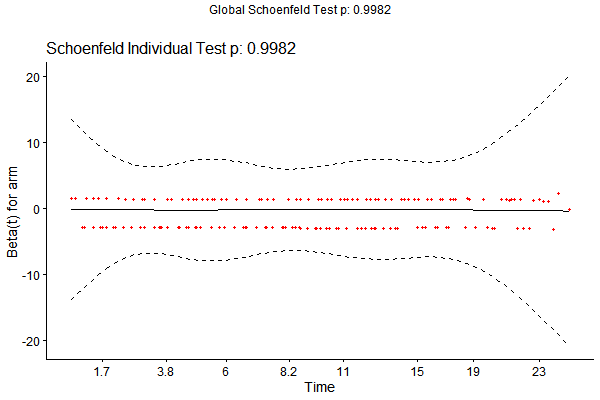 | 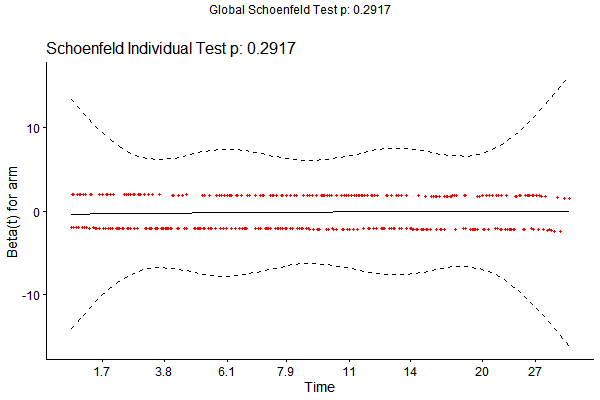 | 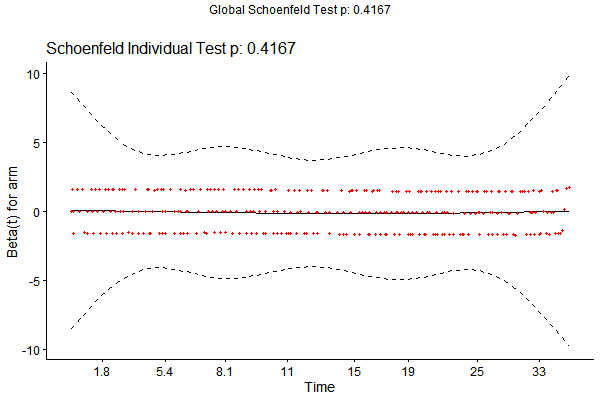 |
| 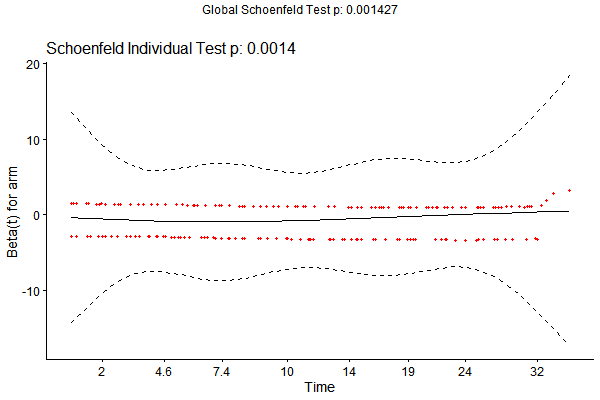 | 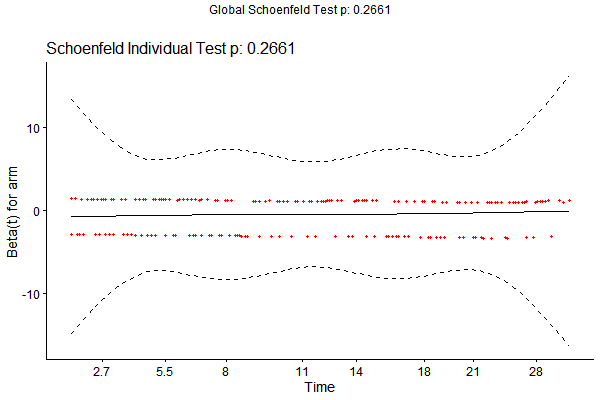 | 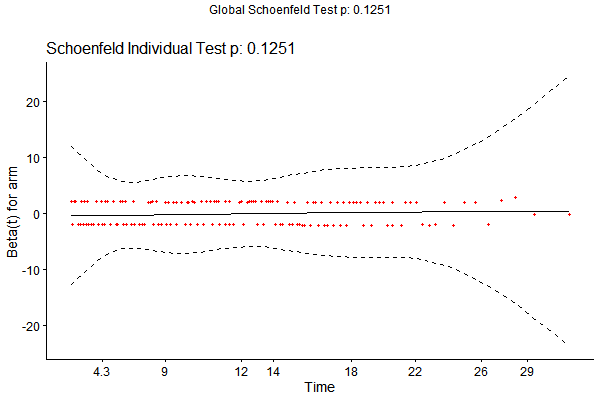 |
| **PFS** | | |
| 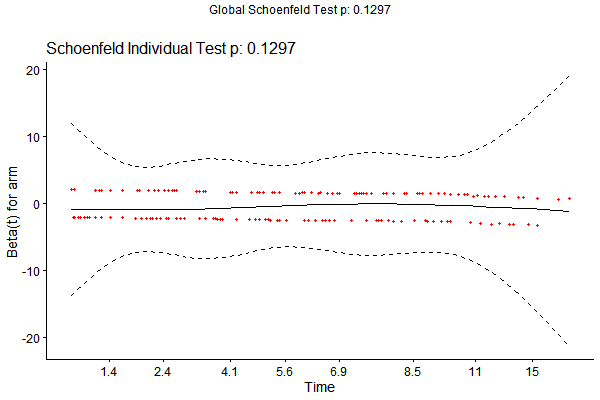 | 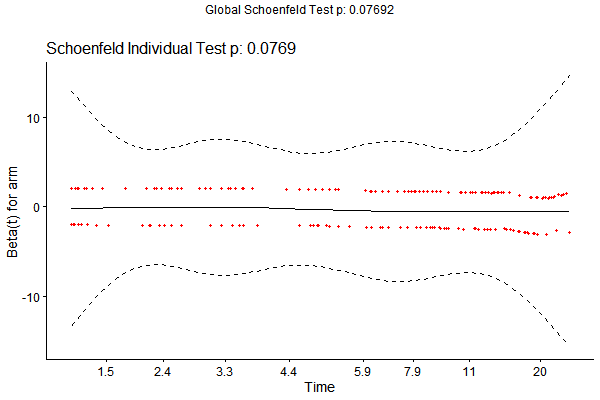 | 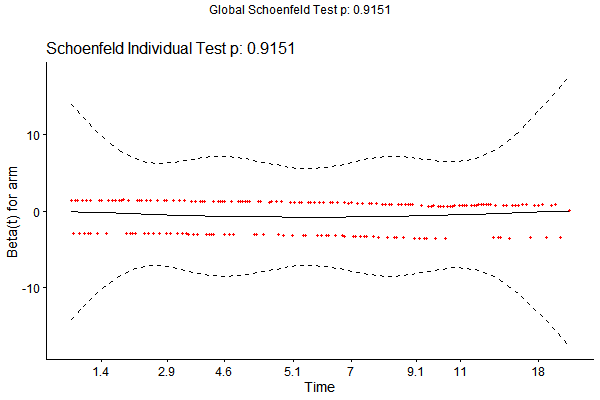 |
| 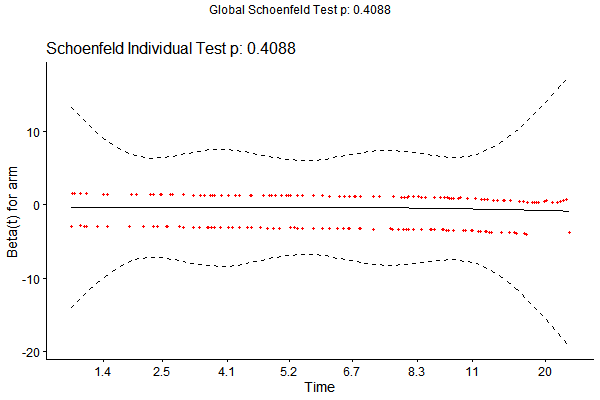 | 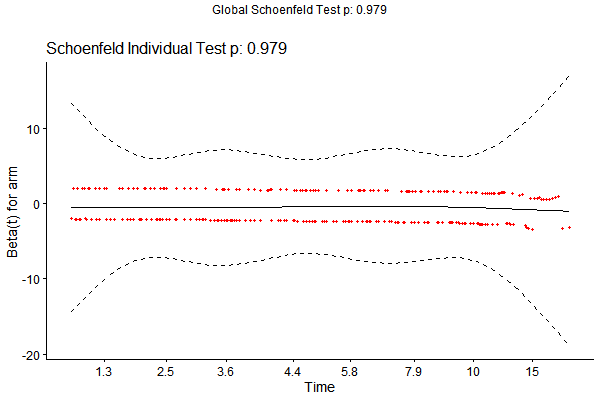 | 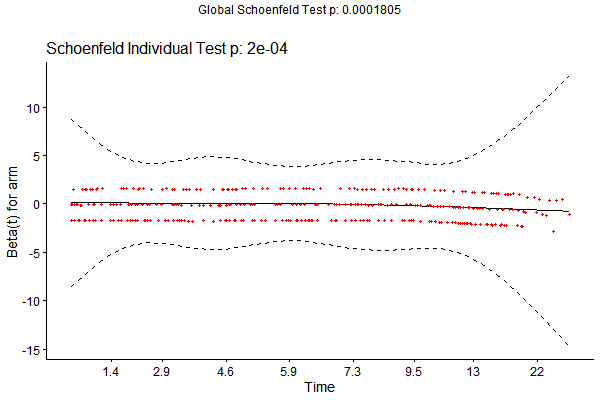 |
| 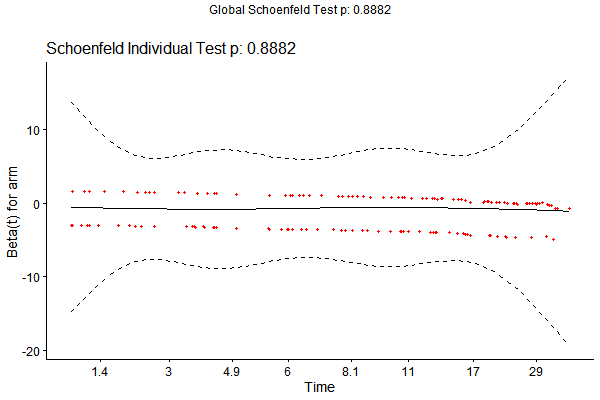 | 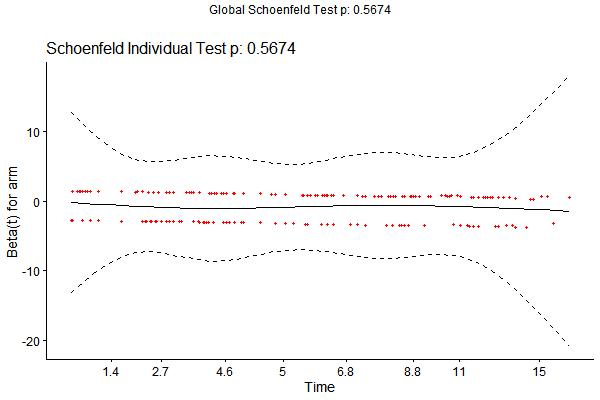 | 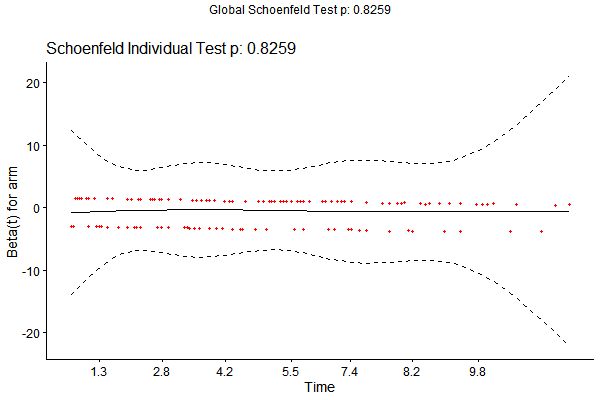 |
| 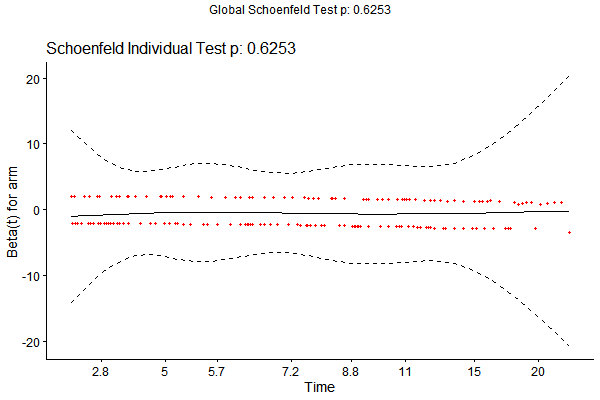 |  |  |
| **OS, PD-L1 expression ≥ 50%** | | |
| 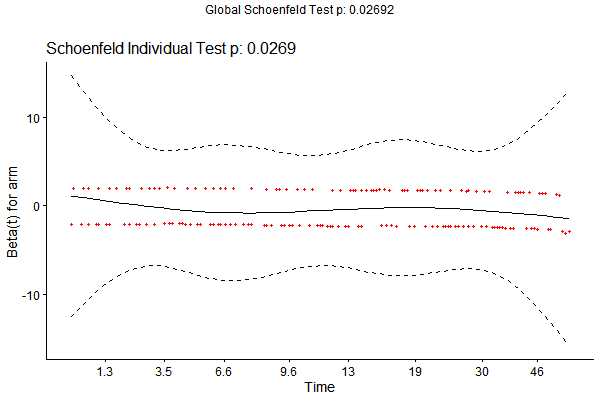 | 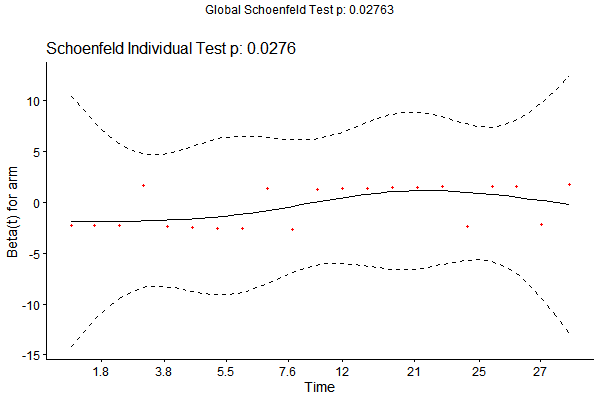 | 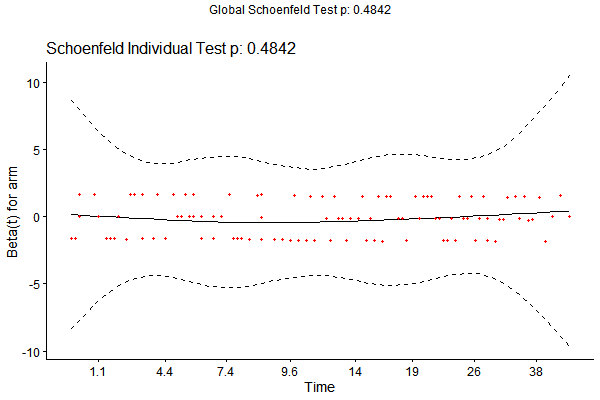 |
| 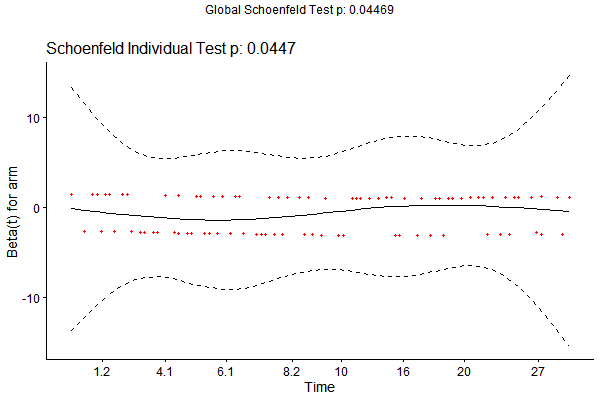 |  |  |
| **OS, PD-L1 expression < 1%** | | |
| 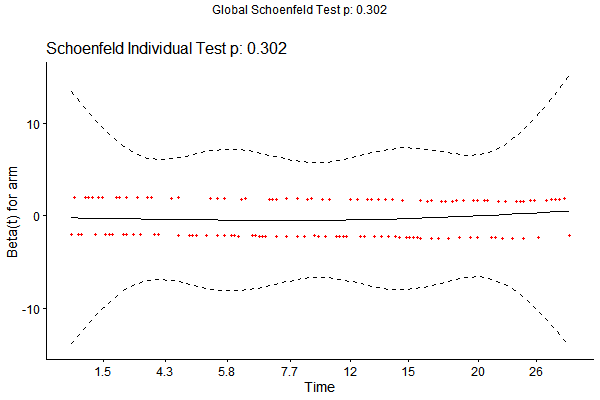 | 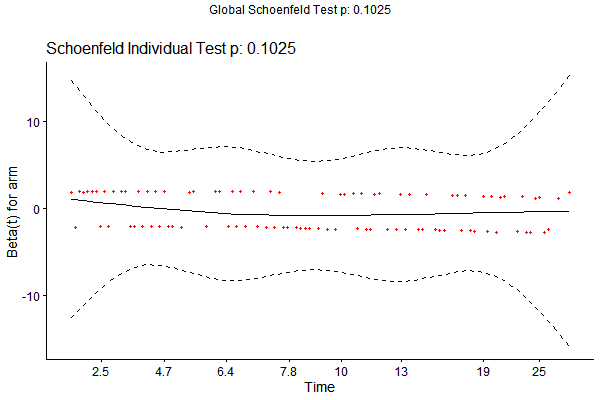 | 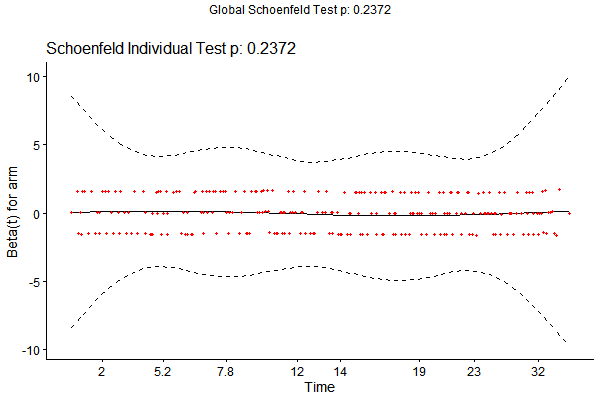 |
| 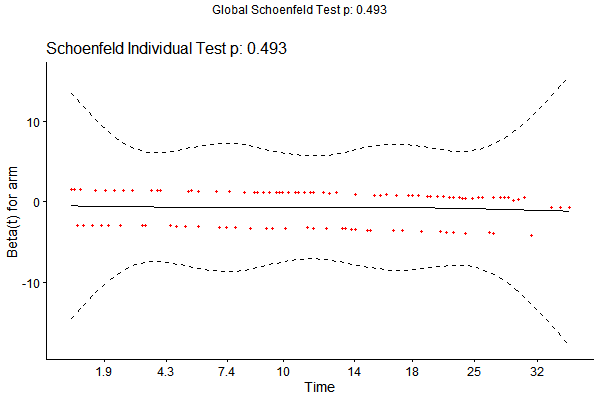 |  |  |
| **PFS, PD-L1 expression ≥ 50%** | | |
| 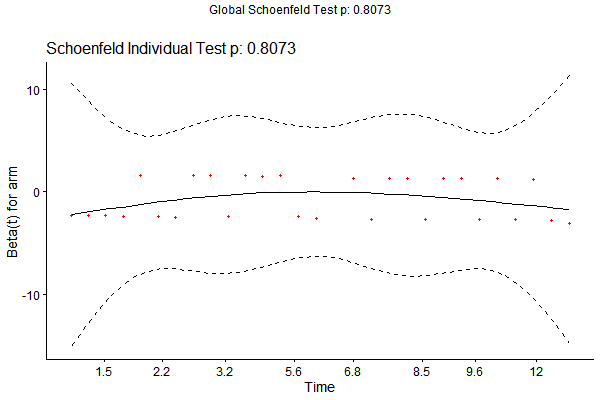 | 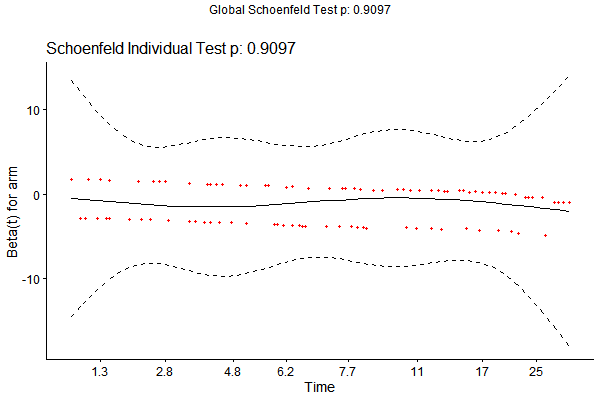 | 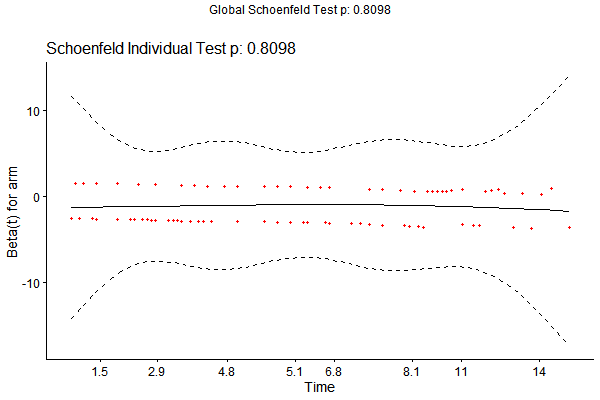 |
| 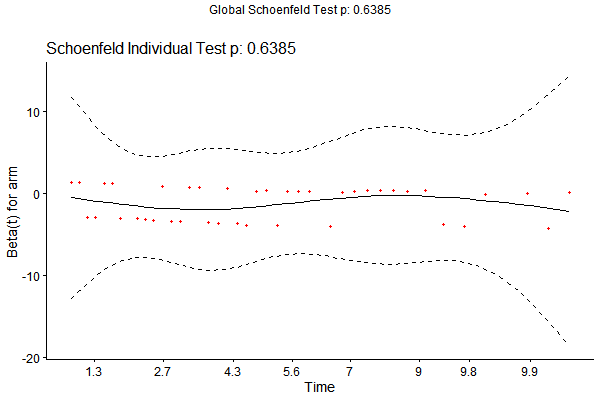 |  |  |
| **PFS, PD-L1 expression < 1%** | | |
| 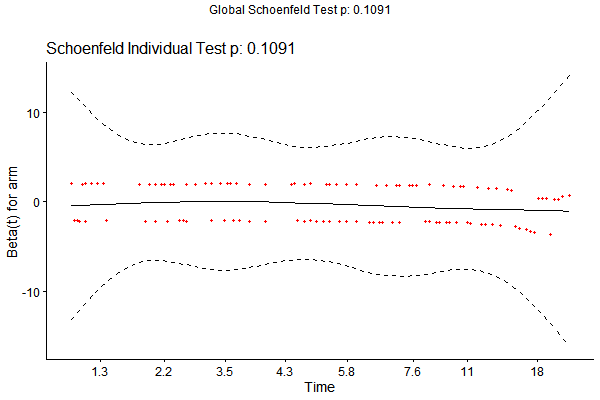 | 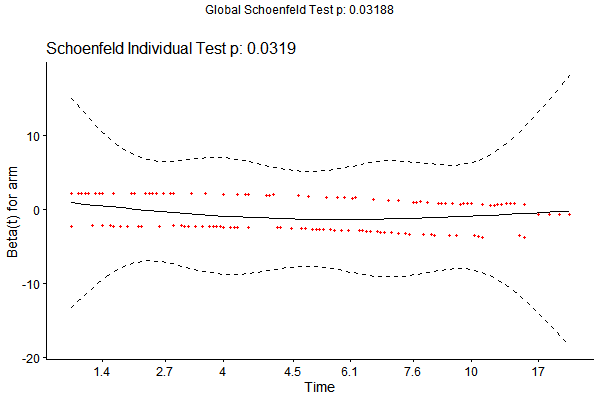 | 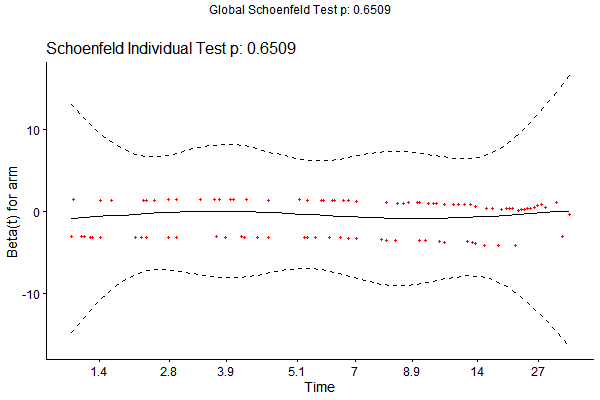 |
| 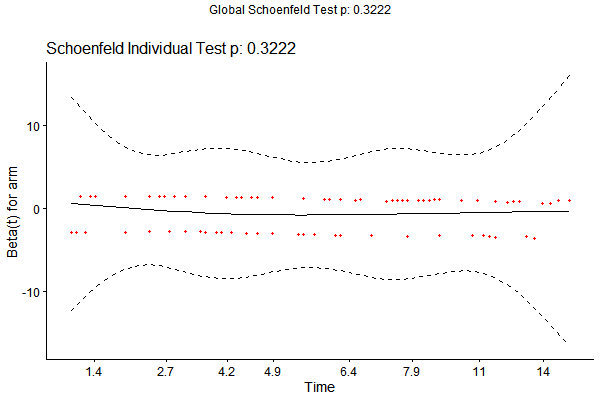 | 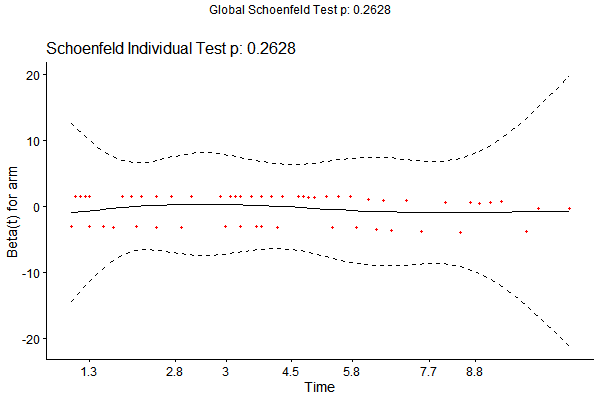 |  |

#### Supplementary Figure 3

| **Figure of risk of bias assessment** |
| --- |
| 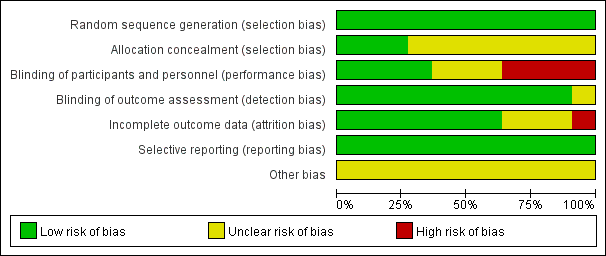 |
| 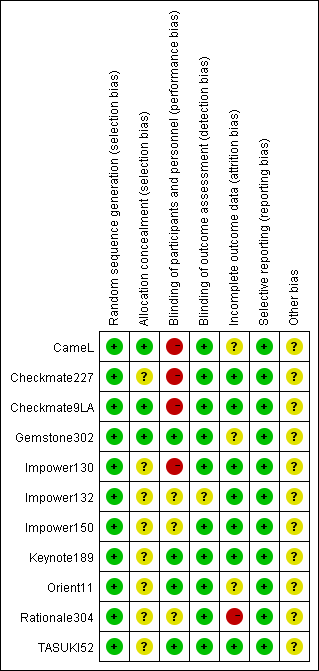 |

#### Supplementary Figure 4

| **Funnel plot to detect the publication bias of included studies** | |
| --- | --- |
| OS  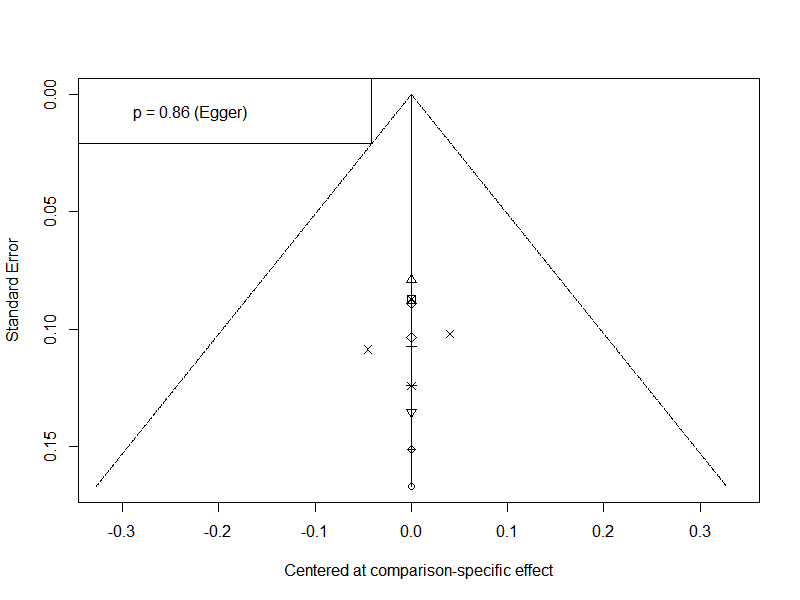 | PFS  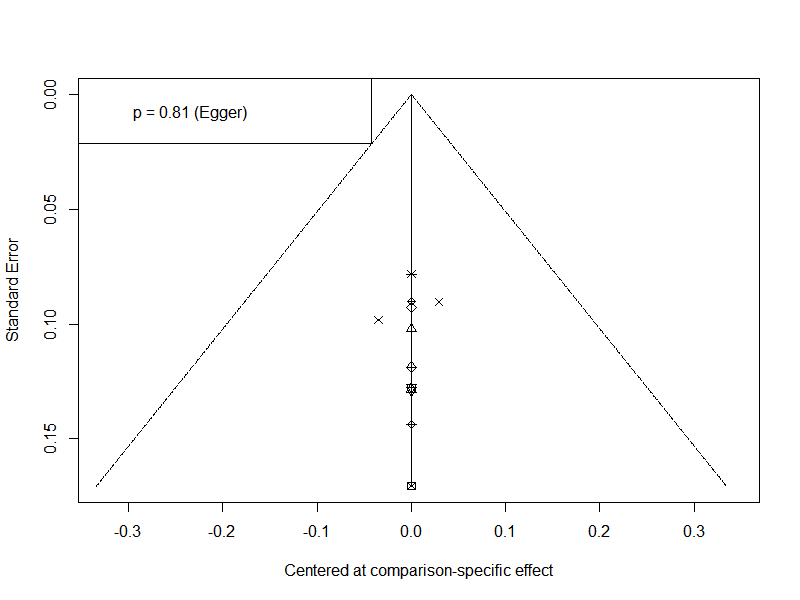 |
| OS: overall survival; PFS: progression-free survival | |

#### Supplementary Figure 5

| **Forest plot of safety** |
| --- |
| 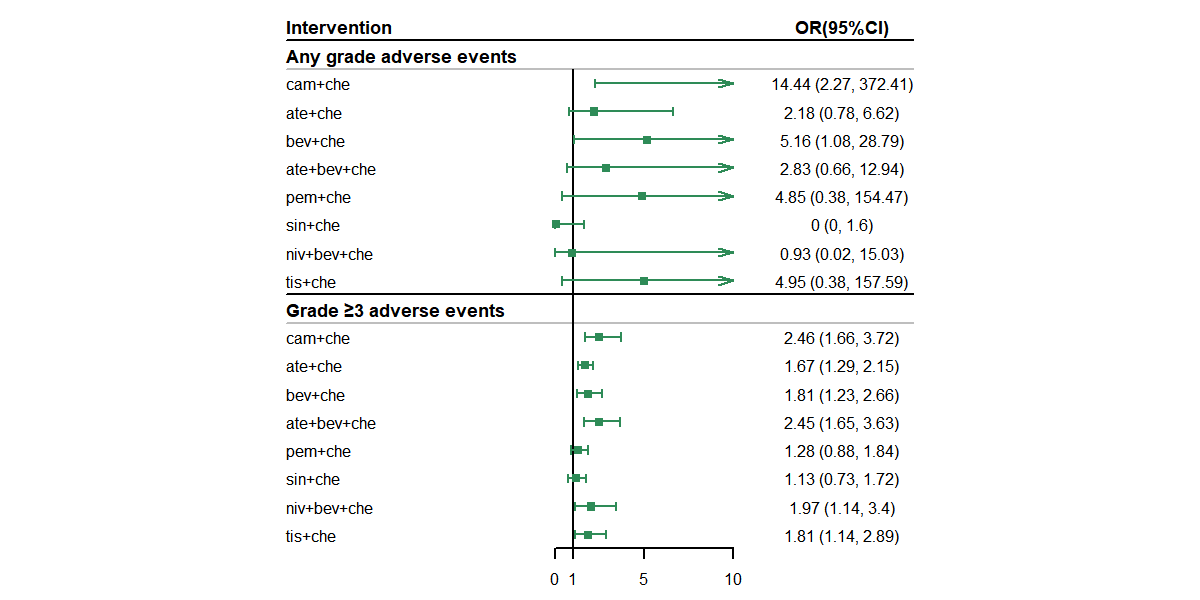 |
| Che: chemotherapy; ate+che: atezolizumab plus chemotherapy; cam+che: camrelizumab plus chemotherapy; ate+bev+che: atezolizumab plus bevacizumab plus chemotherapy; bev+che: bevacizumab plus chemotherapy; niv+bev+che: nivolumab plus bevacizumab plus chemotherapy; pem+che: pembrolizumab plus chemotherapy; tis+che: tislelizumab plus chemotherapy; sin+che: sintilimab plus chemotherapy; OS: overall survival; PFS: progression-free survival; OR: Odds ratio. Reference treatment arm in this forest plot is chemotherapy. |

#### Supplementary Figure 6

| **Survival plot of RP models for subgroup analysis of PD-L1 expression** |
| --- |
| 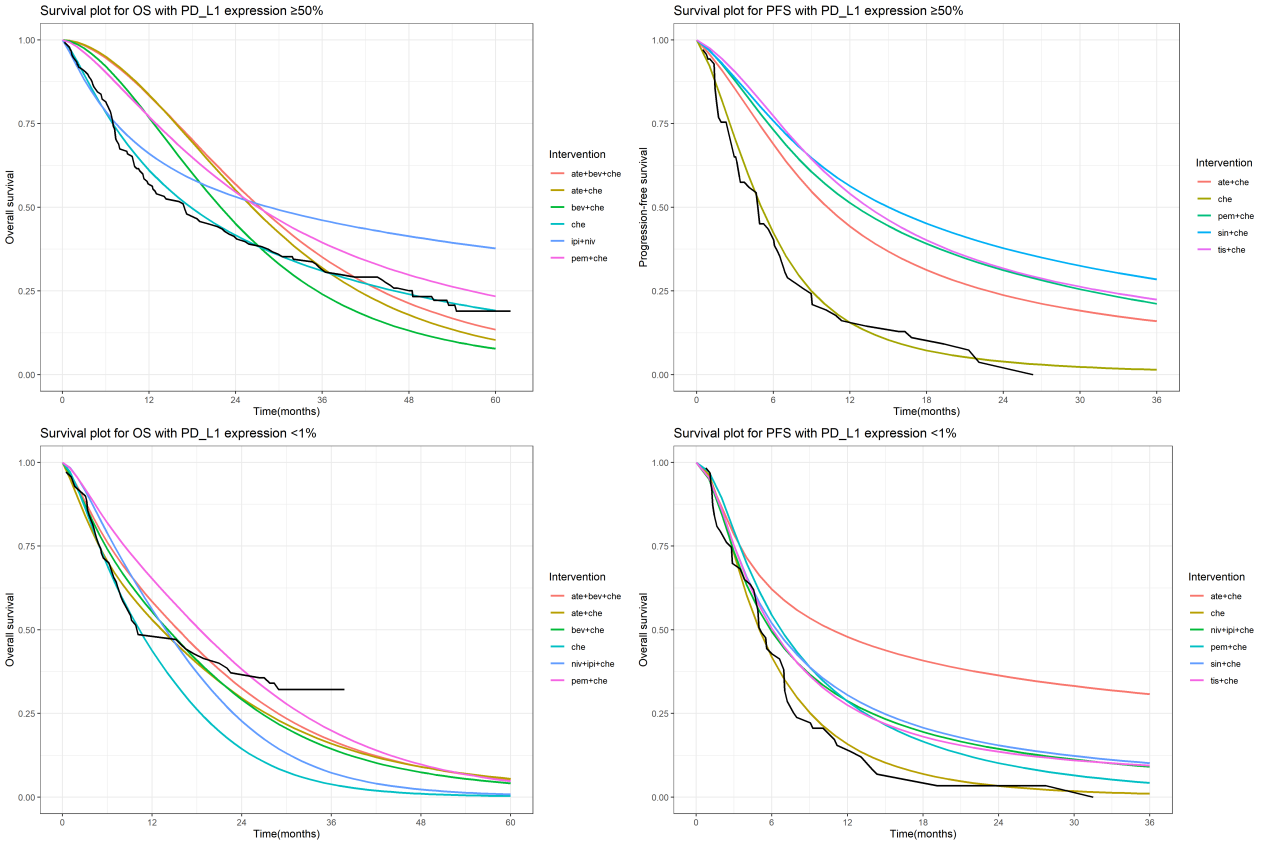 |
| Che: chemotherapy; niv+ipi: nivolumab plus ipilimumab; ate+che: atezolizumab plus chemotherapy; cam+che: camrelizumab plus chemotherapy; niv+ipi+che: nivolumab plus ipilimumab plus chemotherapy; ate+bev+che: atezolizumab plus bevacizumab plus chemotherapy; bev+che: bevacizumab plus chemotherapy; pem+che: pembrolizumab plus chemotherapy; tis+che: tislelizumab plus chemotherapy; sin+che: sintilimab plus chemotherapy; OS: overall survival; PFS: progression-free survival. Black line in survival plots indicated the KM curves of the reference chemotherapy. |

#### Supplementary Figure 7

| **Rank plot of RP models for subgroup analysis of PD-L1 expression** |
| --- |
| 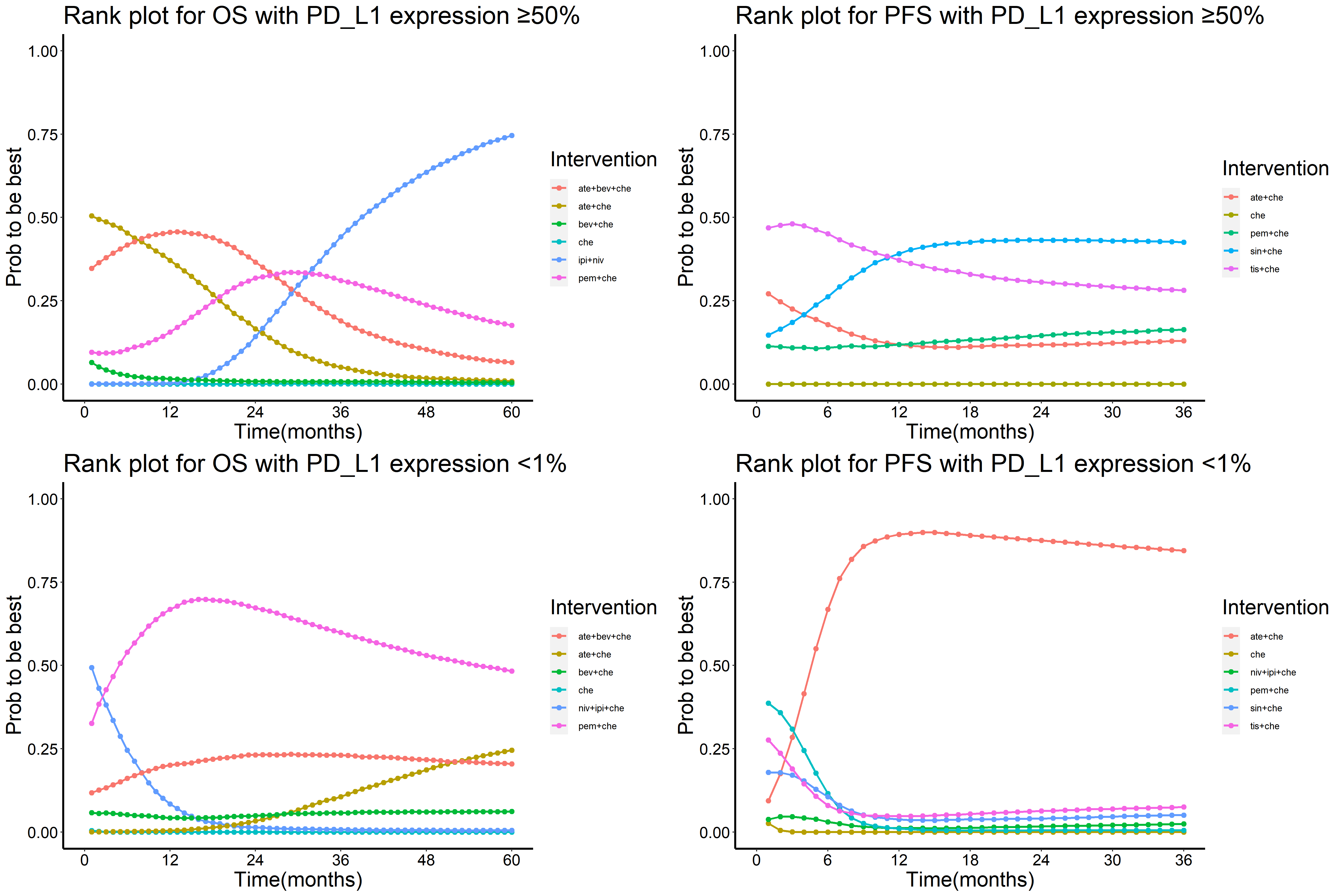 |
| Che: chemotherapy; niv+ipi: nivolumab plus ipilimumab; ate+che: atezolizumab plus chemotherapy; cam+che: camrelizumab plus chemotherapy; niv+ipi+che: nivolumab plus ipilimumab plus chemotherapy; ate+bev+che: atezolizumab plus bevacizumab plus chemotherapy; bev+che: bevacizumab plus chemotherapy; pem+che: pembrolizumab plus chemotherapy; tis+che: tislelizumab plus chemotherapy; sin+che: sintilimab plus chemotherapy; OS: overall survival; PFS: progression-free survival. Prob to be best: probability to be best at each month during 36/60 months. |

#### Supplementary Figure 8

| **Rank plot of RMST for subgroup analysis of PD-L1 expression** |
| --- |
| 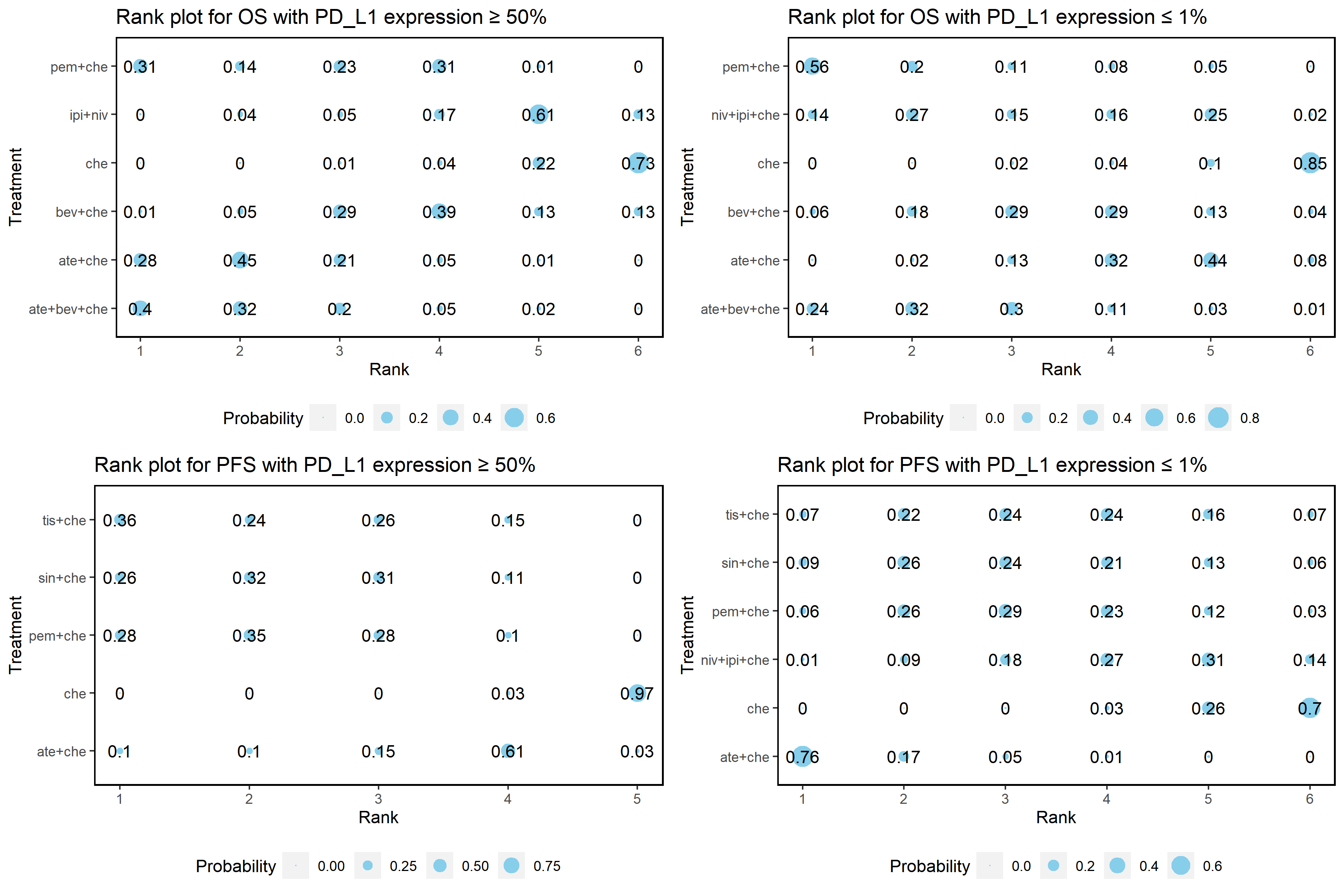 |
| In rank plot, size of each point is proportional to the probability. Che: chemotherapy; niv+ipi: nivolumab plus ipilimumab; ate+che: atezolizumab plus chemotherapy; cam+che: camrelizumab plus chemotherapy; niv+ipi+che: nivolumab plus ipilimumab plus chemotherapy; ate+bev+che: atezolizumab plus bevacizumab plus chemotherapy; bev+che: bevacizumab plus chemotherapy; pem+che: pembrolizumab plus chemotherapy; tis+che: tislelizumab plus chemotherapy; sin+che: sintilimab plus chemotherapy; OS: overall survival; PFS: progression-free survival. Probability: probability to be best at 12/18th month. |

#### Supplementary Figure 9

| **Forest plot of subgroup analysis of NCCN recommended treatments** |
| --- |
| 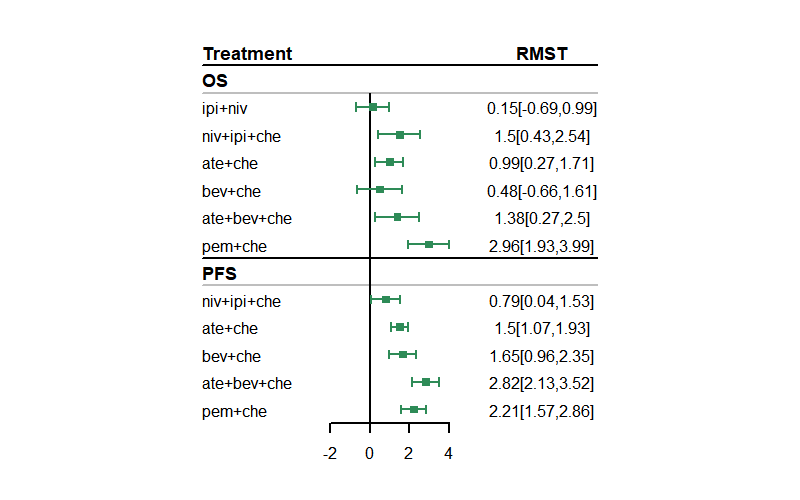 |
| Che: chemotherapy; niv+ipi: nivolumab plus ipilimumab; ate+che: atezolizumab plus chemotherapy; niv+ipi+che: nivolumab plus ipilimumab plus chemotherapy; ate+bev+che: atezolizumab plus bevacizumab plus chemotherapy; bev+che: bevacizumab plus chemotherapy; pem+che: pembrolizumab plus chemotherapy; OS: overall survival; PFS: progression-free survival. |

#### Supplementary Figure 10

| **Survival plots and rank plots for subgroup analysis of NCCN recommended treatments** |
| --- |
| 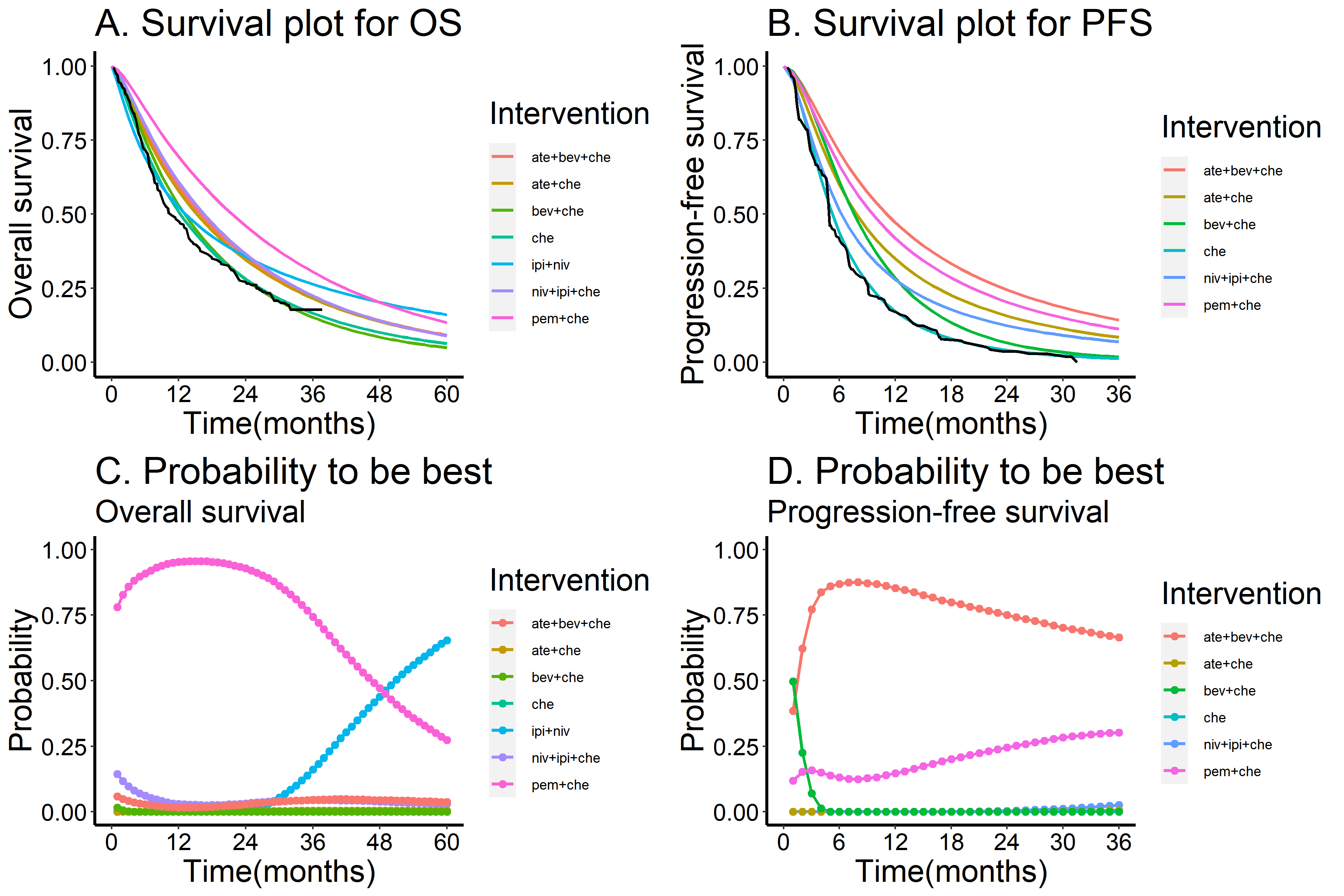 |
| Che: chemotherapy; niv+ipi: nivolumab plus ipilimumab; ate+che: atezolizumab plus chemotherapy; niv+ipi+che: nivolumab plus ipilimumab plus chemotherapy; ate+bev+che: atezolizumab plus bevacizumab plus chemotherapy; bev+che: bevacizumab plus chemotherapy; pem+che: pembrolizumab plus chemotherapy; OS: overall survival; PFS: progression-free survival. Black line in survival plots indicated the KM curves of the reference chemotherapy. Probability: probability to be best at each month during 36/60 months. |

#### Supplementary Figure 11

| **Results of pairwise meta-analysis** |
| --- |
| 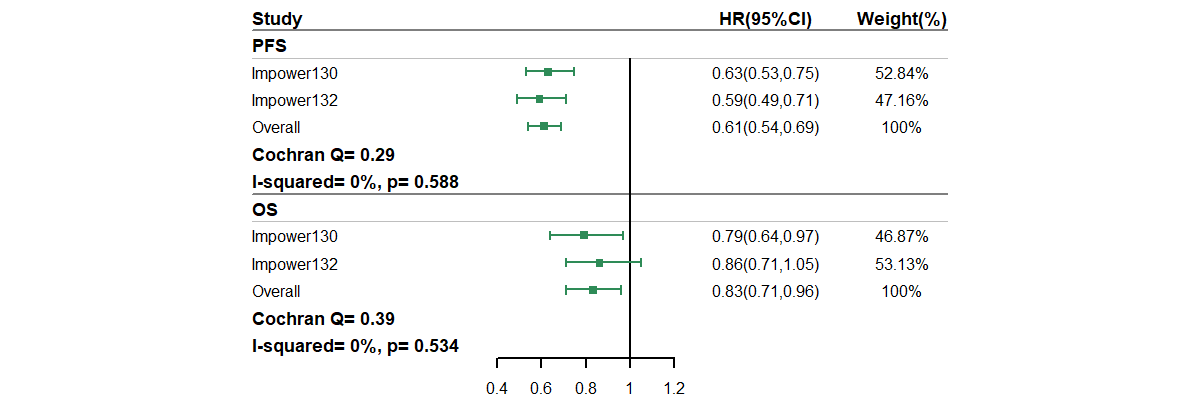 |
| OS: overall survival; PFS: progression-free survival. HR: hazard ratio. |
